# A Randomized Controlled Phase Ib Trial of the Malaria Vaccine Candidate GMZ2 in African Children

**DOI:** 10.1371/journal.pone.0022525

**Published:** 2011-07-28

**Authors:** Sabine Bélard, Saadou Issifou, Aurore B. Hounkpatin, Frieder Schaumburg, Ulysse Ateba Ngoa, Meral Esen, Rolf Fendel, Pablo Martinez de Salazar, Raymund E. Mürbeth, Paul Milligan, Nathalie Imbault, Egeruan Babatunde Imoukhuede, Michael Theisen, Søren Jepsen, Ramadhani A. Noor, Brenda Okech, Peter G. Kremsner, Benjamin Mordmüller

**Affiliations:** 1 Medical Research Unit, Albert Schweitzer Hospital, Lambaréné, Gabon; 2 Institute of Tropical Medicine, University of Tübingen, Tübingen, Germany; 3 Faculty of Epidemiology and Population Health, London School of Hygiene and Tropical Medicine, London, United Kingdom; 4 European Malaria Vaccine Initiative, Statens Serum Institut, Copenhagen, Denmark; 5 Department of Clinical Biochemistry and Immunology, Statens Serum Institut and Centre for Medical Parasitology at the Department of International Health, Immunology and Microbiology, University of Copenhagen, Copenhagen, Denmark; 6 Statens Serum Institut, Copenhagen Denmark; 7 African Malaria Network Trust, Dar es Salaam, Tanzania; Burnet Institute, Australia

## Abstract

**Background:**

GMZ2 is a fusion protein of *Plasmodium falciparum* merozoite surface protein 3 (MSP3) and glutamate rich protein (GLURP) that mediates an immune response against the blood stage of the parasite. Two previous phase I clinical trials, one in naïve European adults and one in malaria-exposed Gabonese adults showed that GMZ2 was well tolerated and immunogenic. Here, we present data on safety and immunogenicity of GMZ2 in one to five year old Gabonese children, a target population for future malaria vaccine efficacy trials.

**Methodology/Principal Findings:**

Thirty children one to five years of age were randomized to receive three doses of either 30 µg or 100 µg of GMZ2, or rabies vaccine. GMZ2, adjuvanted in aluminum hydroxide, was administered on Days 0, 28 and 56. All participants received a full course of their respective vaccination and were followed up for one year. Both 30 µg and 100 µg GMZ2 vaccine doses were well tolerated and induced antibodies and memory B-cells against GMZ2 as well as its antigenic constituents MSP3 and GLURP. After three doses of vaccine, the geometric mean concentration of antibodies to GMZ2 was 19-fold (95%CI: 11,34) higher in the 30 µg GMZ2 group than in the rabies vaccine controls, and 16-fold (7,36) higher in the 100 µg GMZ2 group than the rabies group. Geometric mean concentration of antibodies to MSP3 was 2.7-fold (1.6,4.6) higher in the 30 µg group than in the rabies group and 3.8-fold (1.5,9.6) higher in the 100 µg group. Memory B-cells against GMZ2 developed in both GMZ2 vaccinated groups.

**Conclusions/Significance:**

Both 30 µg as well as 100 µg intramuscular GMZ2 are immunogenic, well tolerated, and safe in young, malaria-exposed Gabonese children. This result confirms previous findings in naïve and malaria-exposed adults and supports further clinical development of GMZ2.

**Trial Registration:**

ClinicalTrials.gov NCT00703066

## Introduction

Malaria control relies primarily on case management and the use of impregnated bednets. Recently, a decline in the incidence of malaria associated with scaling up of interventions has been reported in many countries [1], raising hopes that malaria could be eliminated in these countries [2]. However, before these goals can be achieved, significant political, economic as well as scientific hurdles need to be addressed. Malaria vaccines may have an important role in malaria control and elimination, in part because they can be readily integrated into existing health care structures. Two main lines of research on malaria vaccines have resulted in advanced clinical development programmes, the induction of immunity against pre-erythrocytic stages, and mimicry of naturally acquired immunity to clinical malaria through vaccination with blood stage antigens. GMZ2 is one representative of the second group which is currently under clinical development and RTS,S/AS01 is the most advanced of the first group [3]. Naturally acquired immunity is not sterile but protects from severe symptoms and complications, and despite marked genetic and antigenic variability of the parasite, is remarkably robust. Several passive transfer experiments showed that antibodies are central to the development of natural immunity [4], [5], [6]. Robustness of naturally-acquired immunity was most convincingly demonstrated when antibody preparations from one continent (Africa) were used to inhibit *in vivo* growth of parasites in malaria patients from another continent (Asia) [5]. Unfortunately, the mechanism of protection and the targets of immunity are not known although several immunological surrogate markers of protection have been proposed. Analysis of sera from immune adults from endemic regions, including those from passive transfer experiments, led to the identification of merozoite surface protein 3 (MSP3) as a potentially important antigen for the induction of anti-malarial immunity [7]. Another antigen that elicits antibodies in frequently exposed individuals is glutamate rich protein (GLURP). High anti-GLURP titers have been associated with protection from malaria in several studies [8], [9] and it was shown that anti-GLURP antibodies induce antibody-dependent cellular inhibition (ADCI) in a way similar to MSP3 [10]. A fusion protein of conserved parts of both proteins showed good immunogenicity. Since the presumed mechanism of protection of MSP3 and GLURP is similar, and partial protection in pre-clinical trials was documented [11], it was decided to develop a MSP3-GLURP fusion protein for studies in humans naturally exposed to malaria.

GMZ2 is the preparation of a recombinant GLURP_27–500_-MSP3_212–380_ fusion protein, expressed in *Lactococcus lactis*, and mixed with the adjuvant aluminium hydroxide. The vaccine is given intramuscularly as three injections one month apart. The first trial in humans, in German malaria-naïve adults, showed good safety, tolerability and immunogenicity of three different dosing schemes of GMZ2 [12]. Subsequently, a randomized controlled trial in malaria-exposed Gabonese adults was done, where the higher dose of GMZ2 (100 µg) was compared to a control vaccine (rabies vaccine). In this study, the vaccine was well tolerated and boosted pre-existing immune responses against the vaccine antigen [13]. The vaccine induced strong responses to GMZ2 and to GLURP, but responses to MSP3 were low. The present trial was designed to provide first data on safety and immunogenicity of 100 µg GMZ2, and a lower dose 30 µg GMZ2, in malaria-exposed children 1–5 years of age, the group in which phase IIb efficacy trials would be done.

## Methods

The protocol for this trial and supporting CONSORT checklist are available as supporting information; see Checklist S1 and Protocol S1.

### Participants and interventions

The study took place from September 2008 to October 2009 at the Albert Schweitzer Hospital Lambaréné, Gabon, an area with year-round malaria transmission [14]. Healthy children from Lambaréné between 1 and 5 years of age were screened and those who met the inclusion criteria were randomly assigned 1 1 1 to receive three doses of either rabies vaccine, 30 µg GMZ2 (GMZ2-30) or 100 µg GMZ2 (GMZ2-100). Vaccine doses were given one month apart (days 0, 28 and 56) intramuscularly, alternately in the left or right deltoid muscle. GMZ2 was produced as one clinical batch following Good Manufacturing Practice (Henogen SA, Belgium) and provided in aliquots of 12 µ. Aluminium hydroxide (Statens Serum Institut, Denmark) was provided in separate vials and mixed with the vaccine one hour before administration following a standard operating procedure in a separate preparation room equipped with a sterile flow hood. Rabies vaccine (Verorab, Sanofi Pasteur) was used as control vaccine. Each child was kept under observation for 30 minutes after vaccination. A physician examined participants on the day of vaccination and 1, 3, 7, and 14 days after each vaccination. Participants were re-examined by a physician on Day 84 (one month after the last dose of vaccine) and on Day 365 (10 months after the last vaccine dose). Children were visited at home by a field worker 2, 4, 5 and 6 days after each vaccination and on days 140, 224 and 308 after the first vaccination to assess health and record the occurrence of adverse events. A 24-hour phone line was maintained for parents to contact the study team in the event of any adverse event.

### Ethics

Written informed consent was obtained from the parents of participating children. If the child's parent or guardian was unable to read, an impartial witness was present during the informed consent and signed together with the child's parent the informed consent form. The study was approved by the Comité d'Ethique Régional Indépendant de Lambaréné (CERIL) and the Gabonese Ministry of Health. A data and safety monitoring board (DSMB) monitored subject safety during the trial. The study was conducted according to the Declaration of Helsinki (5^th^ revision) and International Conference on Harmonization Good Clinical Practice (ICH-GCP) guidelines.

### Objectives and outcomes

The primary objective was to evaluate the safety and reactogenicity of three injections of either 30 or 100 µg GMZ2 compared to rabies vaccine in the target group for a clinical phase IIb efficacy trial. As secondary objective, humoral immune responses were assessed.

### Randomization and blinding

A screening list of 39 eligible individuals was sorted on sex and age; 30 were required for enrolment, so 9 individuals (every 4^th^ individual in the sorted list) were excluded to be used as reserves in case any selected individuals dropped out before the administration of the first vaccine dose. The 30 individuals in the sorted list were then allocated to vaccine group using randomly permuted blocks of 3 individuals per block. If a subject was withdrawn before vaccine dose 1, they were to be replaced with the reserve nearest in age. If a subject was withdrawn after vaccine dose 1, they were not replaced. The randomization list was sent by electronic mail by a study statistician (PM) to the study pharmacist who prepared sealed envelopes. A separate sealed envelope of the randomization codes was kept by the local safety monitor. The study pharmacists prepared the vaccines in identical opaque syringes; they had no other role in the trial.

### Laboratory analyses

Immunological assays were done as previously described [12], [13] with minor modifications. For antibody measurement, enzyme linked immunoassays (ELISA) were done. Antigen-specific antibody concentration was assessed by solving a four-parameter logistic regression equation fitted to a serial dilution of a serum pool of highly positive adults according to published procedures [15]. Therefore antibody data is expressed as fraction of antibody concentration compared to a positive serum pool prepared from high responders of a previous clinical trial on GMZ2 [13]. Memory B-cell enzyme linked immunospot assays (ELISPOT) were done on freshly isolated peripheral blood mononuclear cells using previously published procedures [12], [13]. Anti-GMZ2, anti-MSP3, and anti-GLURP antibodies were measured by ELISA, whereas memory B-cell response was assessed by ELISPOT against the whole vaccine antigen (GMZ2) and anti-IgG as a positive control.

### Statistical methods and sample size

The aim of the trial was to establish safety of the vaccine doses with respect to very common adverse reactions. In the previous two clinical trials [12], [13] analysis of ten individuals per group sufficed to detect increases in anti-GMZ2 immune responses. Data was captured on paper case report forms and transferred to an electronic database. All analyses were done using Stata v10 and R v2.12.0. Data analysis followed a statistical analysis plan that was finalized before unblinding.

For analysis of antibody concentrations, zero values were considered as left-censored observations, and replaced with a constant value equal to the smallest non-zero concentration for that antigen in the dataset. For each antigen, the ratio of the geometric mean concentration at each time point to the mean concentration pre-vaccination, was calculated with a 95% confidence interval. Analysis of covariance was used to compare humoral responses between the vaccine groups at each time point, with adjustment for the pre-dose-one value as a covariate, after transforming to logarithms, and the adjusted difference in means between vaccine groups was presented as a ratio in the original measurement scale, with a 95% confidence interval.

As sensitivity analysis the log-transformed area under the curve (AUC) of antigen-specific IgG concentration against time was calculated for each subject from day 0 to day 84 and compared between vaccine groups. This provides a single test for each antigen to determine whether the vaccine induced an antibody response. ELISPOT data are given as number of GMZ2-specific cells per total number of antibody secreting cells (ASC). Non-parametric methods were used to calculate difference in AUC between groups in a hierarchical approach: Kruskal-Wallis test for comparison of the three intervention groups and an exact version the Wilcoxon-Mann-Whitney test for pairwise comparisons if the first test rejected the null hypothesis. An estimate of the shift in location and its 95% confidence interval was calculated using the method of Bauer [16]. Solicited local adverse events (pain, swelling, induration, erythema, or pruritis at the injection site, or contralateral reaction) and general adverse events (fever, irritability, drowsiness, loss of appetite, or diarrhoea) were graded as mild, moderate or severe following the guidelines of the Brighton Collaboration [17]. The number of local and general adverse events, and the number of non-solicited adverse events, within 14 days after each dose was tabulated for each vaccine group. Analyses were by intention to treat.

## Results

### Participants flow and recruitment

**Figure 1:**
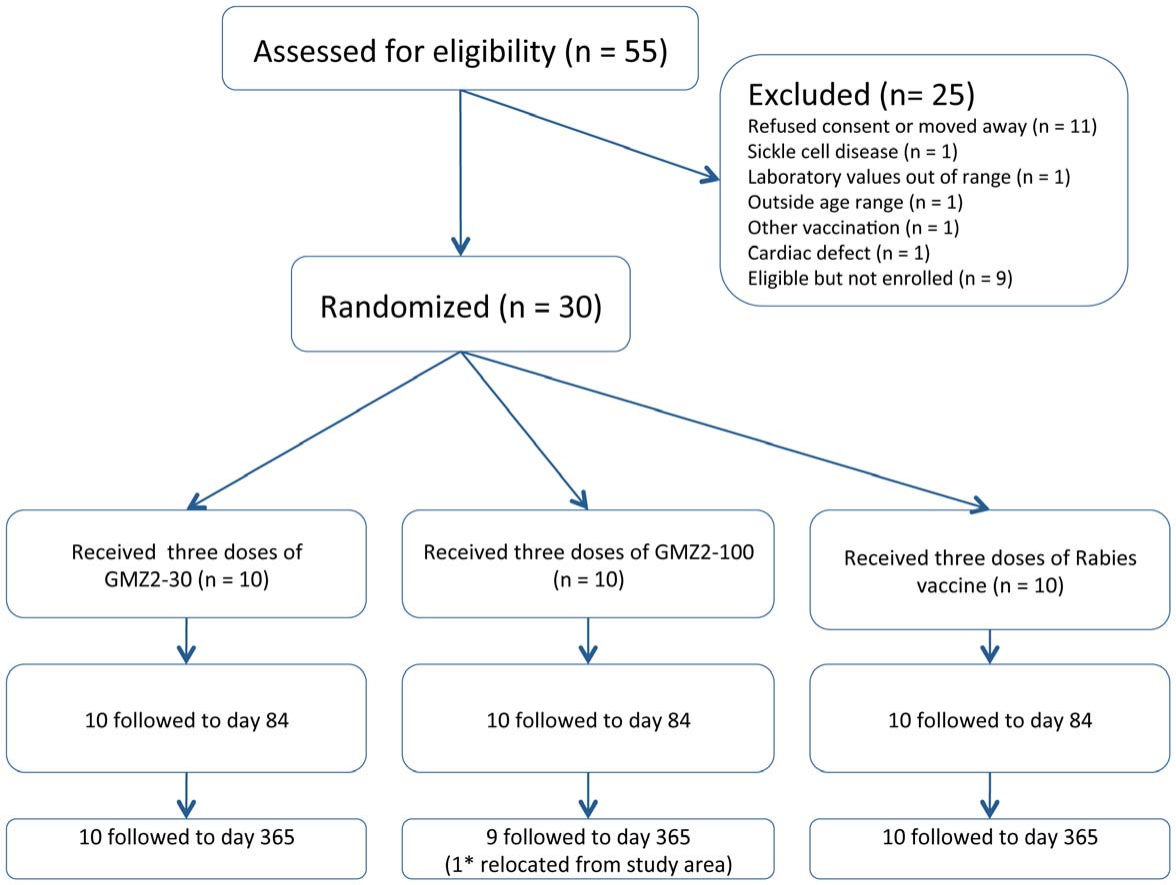
Study flow. All participants received the complete course of vaccination and were followed for 4 weeks after the last vaccination. * The subject lost to follow-up and was in good health when examined after the scheduled Day 365 visit.

Thirty-nine out of 55 children were eligible to participate in the study (Figure 1). The first subject was enrolled on 13^th^ October 2008 and the last patient's follow-up ended 14^th^ October 2009. All participants received their scheduled vaccinations (scheduled on Days 0, 28 and 56) and were closely monitored up to one month after the third dose (Day 84), and follow-up continued until Day 365. One participant of the GMZ2-100 group, a 3-year-old boy, was lost to follow-up at Day 365 due to his relocation from the area. However, the boy was visited and examined two months after the intended last clinical visit. His parents did not report any possible drug-related adverse reaction and he was in good health.

At baseline, participants had similar demographic and laboratory characteristics (Table 1). One participant, a 2-year-old boy, received tetanus vaccine on Day 76 of the study, was excluded from according to protocol analyses of immunogenicity (not shown). Nevertheless, the full set of 30 children was considered for safety and immunological outcomes until Day 84. For analyses including Day 365, all successfully followed 29 children were assessed.

**Table 1:** Baseline characteristics of study participants.

	Rabies	GMZ2-30	GMZ2-100
Age (years) *	3.5 (1.9, 4.8)	3.5 (2.2, 5.6)	3.5 (1.8, 5.7)
Gender #	5/5	5/5	5/5
Height (cm) *	96 (85, 105)	94 (82, 114)	92 (77, 109)
Weight (kg) *	14.6 (11.4, 18.6)	13.7 (11.0, 21.8)	13.3 (9.6, 17.0)
Hemoglobin (g/dl) *	10.3 (9.3, 11.9)	10.5 (8.6, 11.8)	10.5 (8.7, 11.8)
White blood cells (cells/nl)*	10.9 (8.3, 13.6)	9.6 (7.3, 13.4)	8.6 (6.1, 11.4)
Thrombocytes (cells/nl)*	387 (262, 487)	322 (222, 408)	381 (201, 516)

*mean (min, max), # female/male.

### Adverse events

During the trial two serious adverse events (SAE) occurred. A 4-year-old boy was hospitalized because of severe malaria (high parasitemia and prostration) on Day 53. The child recovered without sequelae and received the 3^rd^ vaccination on Day 59. The second SAE occurred in a 3-year-old boy. He presented with fever and upper respiratory infection on Day 29 and was hospitalized for diagnostic purposes on Day 30. The child was discharged in good health on Day 32 without causal treatment and received the third vaccination as planned. Both SAEs occurred in the GMZ2-30 group and were not related to vaccination as judged by the investigators and the DSMB.

**Figure 2:**
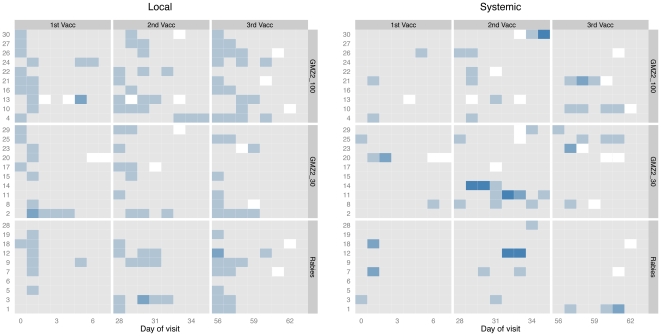
Solicited adverse events. Intensity of local and systemic AE in the 7 days post-vaccination period. Given is the grade of the AE with the highest intensity at each day of follow up as shading (from grey [no AE] to dark blue [grade 3]). Local grade 2 AEs were (top down) pain, swelling, swelling, and pruritus at the injection site. Grade 3 reactions were only present as systemic reactions. They consisted of (top down) fever, fever, loss of appetite, and fever (all fevers were due to upper respiratory infection). All grade 3 AEs were judged not to be related to vaccination. Vaccinations were given on Days 0, 28, and 56.

All children, except for one 4-year-old boy in the rabies vaccine group had either local or systemic reactions during the vaccination period from Day 0 to 2 weeks after the last injection (27 out of 30 with at least one local and 21 out of 30 with at least one systemic reaction). Local and systemic adverse events were slightly more common in the GMZ2-100 group (Figure 2) most of these events were mild in severity. No grade 3 local AE was observed. Within 6 days following vaccination two participants of the rabies and one participant of the GMZ2-100 and -30 groups experienced local grade 2 reactions. Four grade 3 systemic reactions were observed after the second vaccine administration, one case of loss of appetite in the GMZ2-30 group and three cases of fever (≥39°C), one in each intervention group. All grade 3 fever cases were associated with upper respiratory infections and resolved without specific treatment.

Unsolicited adverse events during the trial, were all judged not to be related to the vaccines. In total, 114 unsolicited AE were observed: none of severe intensity, two of moderate and 112 of mild intensity (Table 2). Of note, one 4-year-old boy of the rabies vaccine group developed mild malaria on Day 32 of the study.

**Table 2:** Non-solicited adverse events grouped by organ system, recorded until one month after last vaccination.

	Rabies	GMZ-30	GMZ-100
Ear	3	0	1
Eye	2	1	2
Fever	0	0	2
Gastrointestinal tract	2	2	0
Malaria	1	0	0
Respiratory tract	22	25	22
Skin	3	5	6
Trauma	2	3	10
Urinary tract	0	0	1
TOTAL	35	36	44

All AEs were of mild intensity besides 2 respiratory tract infections of moderate intensity in the GMZ2-30 and Rabies group, respectively. All AEs resolved without sequelae.

### Immunogenicity

**Figure 3:**
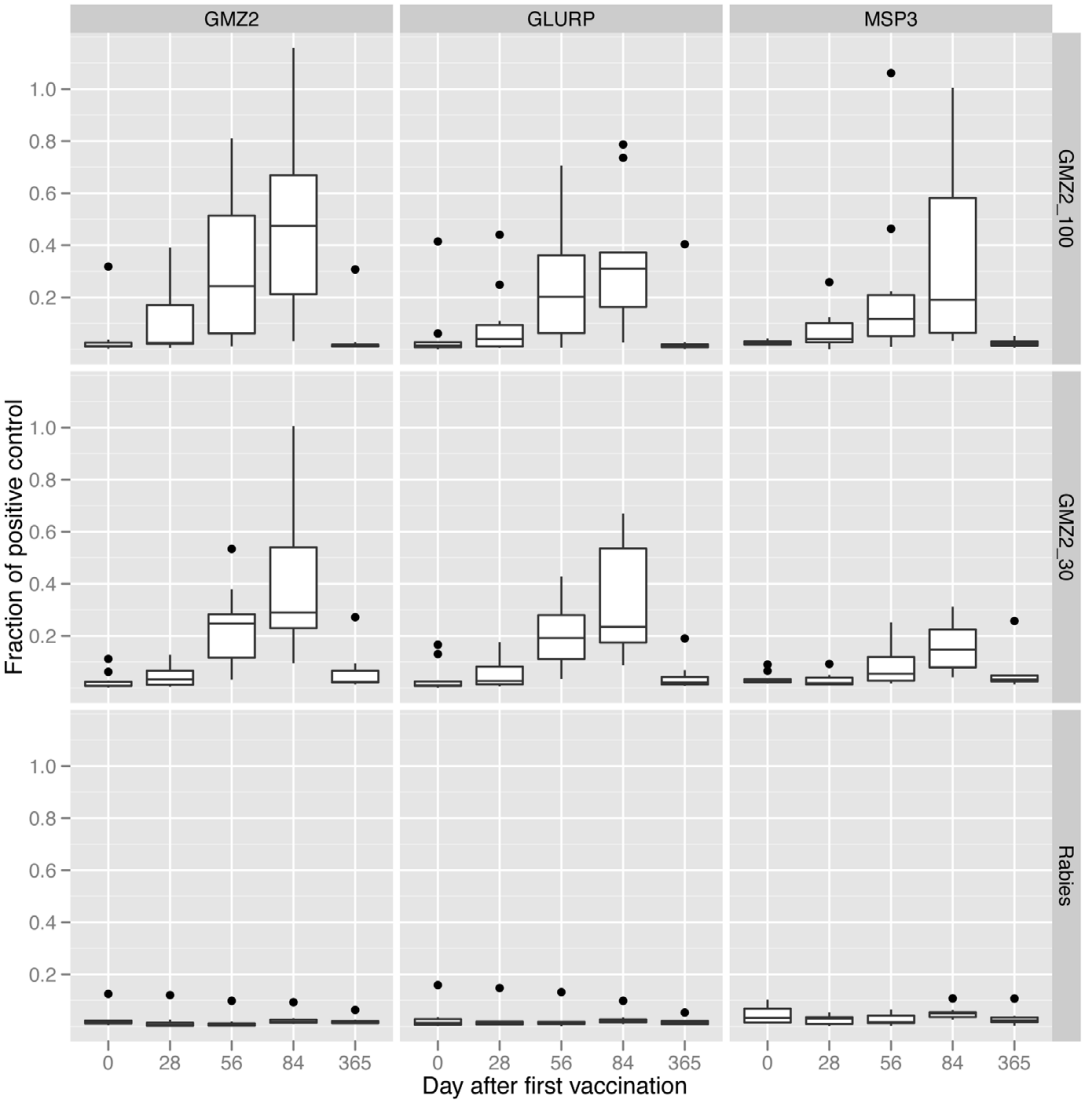
Antibody responses. Antibody responses against GMZ2, GLURP, and MSP3 are shown as boxplot in the original measurement scale (Fraction of positive control).

The concentration of antibodies to GMZ2, GLURP and MSP3 was low in all three groups before vaccination. In both GMZ2 vaccine groups, antibody concentrations to all three antigens increased after each vaccination, with small increases after the first dose and larger increases after the second and third doses (Table 3 and Figure 3). In the GMZ2-30 group, all individuals responded to vaccination with an increase in anti-GMZ2 antibodies at Day 84, whereas in the GMZ2-100 group one non-responder was observed. Confidence intervals for the rises in concentration were wide reflecting the small sample size (Table 3). Anti-MSP3 antibody concentration measured one month after the third dose of vaccine was higher in the GMZ2 vaccinated groups, than in the rabies vaccine group, by 2.7 fold (95%CI: 1.6,4.6) in the GMZ2-30 group and by 3.8 fold (1.5,9.6) in the GMZ2-100 group (ratios adjusted for differences in pre-vaccination antibody concentration). Anti-GLURP antibody concentration was 12 fold higher (7.2,21) in the GMZ2-30 group than in the rabies vaccine group and 9.7 fold higher (4.4,22) in the GMZ2-100 group. Anti-GMZ2 antibody concentration was 19 fold higher (11,34) in the GMZ2-30 group and 16 fold higher (7.4,36) in the GMZ2-100 group.

**Table 3:** Change in antigen-specific antibody concentration to GMZ2, GLURP and MSP3 after 1, 2 and 3 doses of vaccine.

Antigen	GMZ2			GLURP			MSP3		
Vaccine	Rabies	GMZ2-30	GMZ2-100	Rabies	GMZ2-30	GMZ2-100	Rabies	GMZ2-30	GMZ2-100
Day 28	0.12 (0.01,1.1)	2.3 (0.70,7.8)	2.6 (0.74,9.1)	1.1 (0.35,3.1)	2.2 (0.57,8.4)	2.5 (0.56,11)	0.3 (0.07,1.2)	0.46 (0.18,1.2)	0.90 (0.22,3.7)
Day 56	0.21 (0.04,1.0)	17 (6.9,49)	9.5 (2.7,34)	0.90 (0.26,3.1)	13 (3.8,42)	8.6 (1.8,40)	0.50 (0.19,1.3)	1.9 (0.94,3.8)	3.3 (1.2,9.1)
Day 84	1.2 (0.57,2.5)	30 (11,83)	19 (6.1,58)	1.7 (0.62,4.5)	21 (6.4,68)	14 (3.8,56)	1.5 (0.83,2.8)	4.2 (2.4,7.6)	7.1 (2.9,17)
Day 365	1.0 (0.50,2.05)	3.2 (1.5,6.9)	1.1 (0.70,1.8)	1.0 (0.37,2.8)	1.9 (1.1,3.5)	1.0 (0.45,2.2)	0.67 (0.33,1.4)	1.2 (0.71,2.0)	0.83 (0.45,1.5)

Data is given as the ratio of geometric mean IgG concentration (95% confidence interval).

One year after the first vaccination (Day 365) the concentration of anti-GMZ2, anti-MSP3 and anti-GLURP antibodies was similar in the rabies and GMZ2-100 groups while the GMZ2-30 group had a 1.2 (1.1,4.2) fold higher baseline corrected anti-GMZ2 antibody concentration compared to the rabies group on Day 365.

AUCs were calculated to examine if a different approach of analyzing leads to similar conclusions (Table 4). Anti-GMZ2, anti-GLURP and anti-MSP3 antibody AUCs where higher in the GMZ2-30 and GMZ2-100 groups as compared to the rabies vaccinated subjects whereas the GMZ2 groups had similar reactivity, thus confirming the findings of the primary statistical approach.

**Table 4:** Area under the curve IgG concentration.

	Rabies	GMZ2-30	GMZ2-100
Anti-GMZ2	6.7 (4.4,10)	65 (39,109)	58 (24,141)
Anti-GLURP	6.9 (5,11)	53 (32,87)	41 (17,103)
Anti-MSP3	13 (10,18)	29 (18,47)	36 (15,88)

Data is given as geometric mean (95% confidence interval) in fraction of positive control x days.

**Figure 4:**
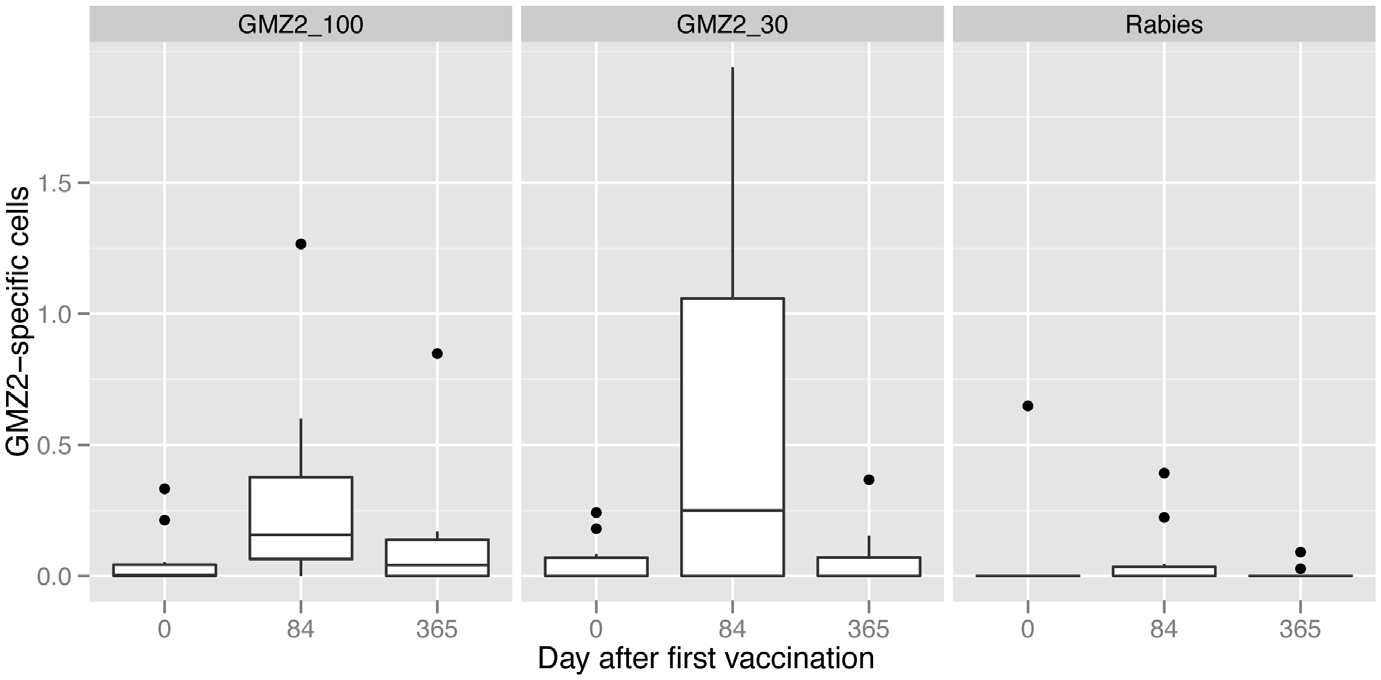
GMZ2-specific memory B-cells. Results are given as GMZ2-specific per 1000 ASCs.

Memory B-cell responses increased upon vaccination with GMZ2 (Figure 4). No increase in anti-GMZ2 memory B-cell numbers at Day 84 was detected in 4 children of the GMZ2-30 and 3 children of the GMZ2-100 groups. AUCs were significantly different between the groups (Kruskal Wallis test; p-value  = 0.008). Pairwise comparison showed a shift in location of 44 (4.2,98) GMZ2-specific spots per ASC times Days for the comparison between rabies and GMZ2-100 AUCs, 49 (7.6,251) for rabies versus GMZ2-30 and a non-significant −7.4 (−211,45) for GMZ2-100 versus GMZ-30. There was no evidence of an association between antigen-specific antibodies and memory B-cell response (data not shown).

## Discussion

Clinical development of malaria vaccines has gained momentum in recent years. Several vaccine candidates have been evaluated in malaria-exposed individuals but only very few have proceeded to efficacy trials in endemic countries. Asexual blood stage vaccines that have been tested for efficacy in naturally exposed individuals (clinical phase IIb) recently, include AMA-1 [18] and MSP1_42_ (FMP1-AS02A) [19]. Unfortunately, these malaria vaccine candidates did not protect children in phase II efficacy trials. Two major difficulties in the selection of potential vaccine antigens are the lack of a surrogate marker for protection and the high degree of variability of antigens that are exposed to the immune system. GMZ2 is one of the latest vaccine candidates to enter clinical trials. Association studies suggest a prominent role of both antigens contained in GMZ2 in protection from malaria and showed partial efficacy in an animal model [11]. In addition, both antigen fragments used in the vaccine are conserved in clinical isolates [20], [21], which may constitute an advantage over other candidates. Therefore a clinical development program for GMZ2 was developed that resulted in the present phase Ib trial to assess safety and immunogenicity in the target population for a subsequent clinical phase IIb efficacy trial.

GMZ2 has been developed under the premise that vaccination schedule should be compatible with the Expanded Program on Immunization (EPI), although it is being increasingly recognized that the EPI scheme may be unlikely to induce maximal immunogenicity. Nonetheless, GMZ2 was well tolerated and induced a robust immune response in its target population. No vaccine-related SAEs or grade 3 AEs were observed and all GMZ2 vaccinated participants developed either anti-GMZ2 antibodies or memory B-cells.

Both the 30 µg and 100 µg GMZ2 induced strong antibody responses to GLURP, as has been seen with GMZ2 in previous trials [12], [13]. Both groups showed responses to MSP3, in contrast to the study in Gabonese adults, where there was no evidence of responses to MSP3. The rise in anti-MSP3 IgG concentration after three doses was somewhat greater in the 100 µg group than in the 30 µg group but the trial was not powered to be able to compare immunogenicity between these two groups, (a much larger trial would have been required to determine whether 30 µg GMZ2 was as immunogenic as 100 µg). Considering data from all phase I trials of GMZ2 [12], [13], the 100 µg dose is safe and well tolerated and was selected for further clinical development, although this does not rule out the use of other doses in the final product. In contrast to malaria-naïve German adults [12], antigen-specific IgG concentrations seemed to decrease by Day 365, but the confidence intervals were wide and the trial was too small to evaluate persistence of IgG response. If anti-parasitic immunity can be induced, it will be important to investigate kinetics of IgG responses and to consider alternative administration schemes, doses, or vaccine formulations to improve longevity of responses and of protection. Such trials will be extraordinarily helpful for understanding immune response patterns and their role in protection.

The number of GMZ2 vaccinated subjects is still low (a total of 70 people have been vaccinated with GMZ2, 40 of them with the 100 µdose), therefore it is too early to assess its overall safety with confidence. Nevertheless, the use of aluminum hydroxide, one of the most frequently administered molecules in vaccinations, as the adjuvant was a choice that minimizes safety problems and therefore the good tolerability is not surprising. We believe that this is an important issue and should be investigated with more emphasis in other vaccines that use novel and experimental adjuvant systems. This is particularly relevant for vaccines intended for widespread use in young children.

In conclusion, GMZ2 is immunogenic and well tolerated. Efficacy studies are now needed and a multi-center phase IIb trial in four countries has been designed to determine whether three doses of 100 µg GMZ2 can protect children from malaria.

## Supporting Information

Checklist S1 (DOC)

Protocol S1 (PDF)
