# Supplementary material for: A Randomized Controlled Phase Ib Trial of the Malaria Vaccine Candidate GMZ2 in African Children
Source: PLoS One. 2011 Jul 28;6(7):e22525. doi: 10.1371/journal.pone.0022525 (PMC3145647; doi:10.1371/journal.pone.0022525)
Supplement: Protocol S1 — (PDF) [file pone.0022525.s002.pdf]

CLINICAL TRIAL PROTOCOL

**Sponsor legal name:**	African Malaria Network Trust
**Finished product**	GMZ2 malaria vaccine
**Active ingredient**	GLURP + MSP 3
**Trial Title:**	A phase I, randomized, controlled, double-blind, single centre trial to evaluate the safety and immunogenicity of 30 and 100 µg of GMZ2 in Gabonese children aged 1-5 years
**Trial Identifier:**	GMZ2_03_08
**Clinical phase**	Phase Ib
**Protocol Version**	Final protocol version 1.7
**Principal Investigator**	Saadou Issifou MD PhD<br>Albert Schweitzer Hospital<br>Medical Research Unit<br>Box: 13901<br>Libreville<br>Gabon<br>Tel: (+241) 07847740
**Sponsor Study Coordination**	African Malaria Network Trust,<br>BOX 33207, Dar es Salaam<br>Tanzania<br>Tel:+255 22 2700018<br>Fax: +255 22 2700380<br>www.amanet-trust.org
**Clinical Development Team**	Roma Chilengi<br>Ibrahim Elhassan<br>Babatunde Imoukhuede<br>Paul Milligan<br>Nathalie Imbault<br>Ramadhani Abdalla Noor<br>Benjamin Mordmüller

STATEMENT OF COMPLIANCE

The study described in this protocol will be conducted according to current International Conference of Harmonization Good Clinical Practice (ICH-GCP) and the applicable regulations of Gabon.

The Regional IRB of Lambaréné (CERIL) and AMANET will review and approve the protocol prior to study start. Documentation of the approval by these bodies will be kept in the PI's study file and a copy in the sponsor study file.

SIGNATURE PAGE

The signatures below constitute the approval of this protocol and the attachments, and provide the necessary assurances that this trial will be conducted according to all stipulations of the protocol, including all statements regarding confidentiality, and according to local legal and regulatory requirements and ICH guidelines.

Research Centre :

Signed: ______________________________ Date: ____________

Director,
Dr. Peter G. Kremsner
Medical Research Unit
Albert Schweitzer Hospital

Site Principal Investigator:

Signed: ______________________________ Date: ____________

Saadou Issifou PhD
Medical Research Unit
Albert Schweitzer Hospital

Sponsor:

Signed: ______________________________ Date: ____________

Wenceslaus L Kilama PhD
Professor

Clinical Trials Coordinator

Signed: ______________________________ Date: ____________

Roma Chilengi BSc MBChB DHTM, MSc

Table of Contents

1 Background Information and Scientific Rationale 12
1.1 Background Information ... 12
1.1.1 Disease burden ... 12
1.1.2 Malaria in Lambaréné (Gabon) ... 12
1.2 Global Epidemiology ... 12
1.3 Prevention and Control of Infection among Humans ... 13
1.4 Malaria vaccines ... 13
1.4.1 GLURP antigen ... 14
1.4.2 MSP3 antigen ... 14
1.5 Name and description of the investigational products ... 15
1.6 Summary of findings from non-clinical studies ... 16
1.6.1 Antigenicity ... 16
1.6.2 Immunogenicity ... 16
1.6.3 Safety and immunogenicity in Saimiri monkeys ... 17
1.7 Summary of previous clinical trials ... 17
1.7.1 Phase Ia trial of MSP3-LSP in “naïve” European adults: ... 17
1.7.2 Phase Ib trial of MSP3-LSP in exposed Burkinabé male adults ... 17
1.7.3 Phase Ia trial of GLURP in “naïve” European adults ... 18
1.7.4 GMZ2 (GLURP-MSP3) Phase I trials ... 18
1.8 Rationale ... 20
1.8.1 Risks and benefit to human participants ... 20
1.8.2 Rationale for adjuvant selection ... 20
1.8.3 Rationale for dosage selection ... 20
1.8.4 Justification for 0, 28, 56 Day Schedule ... 20
1.8.5 Overview of the GMZ 2 clinical development plan ... 21
1.9 Comparator Vaccine: Rabies vaccine ... 21
1.9.1 Rationale for rabies vaccine ... 21
1.9.2 Safety of Rabies vaccine ... 22
1.10 Potential Risks and Benefits ... 22
1.10.1 Potential Risks ... 22
1.10.2 Known Potential Benefits ... 23
2 OBJECTIVES 24
2.1 Primary Objective ... 24
2.2 Secondary Objectives ... 24
2.2.1 Humoral Immune Response ... 24
2.3 Exploratory Objectives ... 24
3 STUDY DESIGN 25
3.1 Overview ... 25
3.2 Site description ... 26
3.3 Participant Inclusion Criteria ... 26
3.4 Participant Exclusion Criteria ... 27
3.5 Treatment Assignment Procedures ... 28

3.5.1 Randomization Procedures 28
3.5.2 Masking Procedures 28
3.5.3 Reasons for Withdrawal 28
3.5.4 Handling of Withdrawals 29
3.5.5 Termination of Study 29
3.5.6 Definitions 29
4 INTERVENTION/INVESTIGATIONAL PRODUCT 31
4.1 Study Product Description 31
4.1.1 Description 31
4.1.2 Formulation 31
4.1.3 Manufacturer 31
4.1.4 Preparation 31
4.1.5 Aluminium hydroxide formulation: 31
4.1.6 Precaution for use 31
4.2 Vaccination 31
4.3 Prior hand Concomitant Therapy 32
4.4 Management of Vaccines- GMZ2 32
4.5 Storage and Shipment Conditions 32
4.6 Accountability 33
4.7 Return of Unused Products 33
4.8 Concomitant Medications/Treatments 33
5 PARTICIPANT PROCEDURES 34
5.1 Screening Day –14 to –1 34
5.2 Enrolment/Baseline 35
5.3 Vaccination process 35
5.4 Follow-up 41
5.5 Unscheduled Visits 42
6 ADVERSE EVENTS AND REPORTING PROCEDURES 43
6.1 Definitions 43
6.1.1 Adverse Event (or Adverse Experience AE) 43
6.1.2 Adverse Drug Reaction (ADR) 43
6.1.3 Unexpected Adverse Drug Reaction 43
6.1.4 Serious Adverse Event 44
6.1.5 Clinical laboratory parameters and other abnormal assessments qualifying as adverse events and serious adverse events 44
6.2 Safety Monitoring Plan 45
6.2.1 Role of the Safety Monitor: 45
6.2.2 The Data Safety Monitoring Board (DSMB) 45
6.2.3 Holding Rules 46
6.2.4 Process for restarting vaccination 46
6.2.5 Process for stopping vaccination of a group or of the trial 46
6.3 Safety Data Collection and Management Procedures 46
6.3.1 Expected Adverse Vaccine Reactions 46

6.3.2 Safety Data Collection ... 47
6.3.3 Time period, frequency, and method of detecting adverse events and serious adverse events ... 48
6.3.4 Assessment of causality ... 49
6.3.5 Medically attended visits ... 50
6.3.6 Follow-up of adverse events and serious adverse events and assessment of outcome ... 50
6.3.7 Reporting of Serious Adverse Events ... 50
6.3.8 Regulatory reporting requirements for serious adverse events ... 51
6.3.9 Post study adverse events and serious adverse events ... 52
6.3.10 Treatment of adverse events ... 52
6.4 Lost to Follow-up Procedures ... 52
7 EVALUATION CRITERIA 53
7.1 Primary Evaluation Criterion: Safety ... 53
7.1.1 Definition of the Criterion ... 53
7.1.2 Parameters to be measured ... 53
7.1.3 Method and Timing of Measurement ... 54
7.2 `Secondary Evaluation Criteria: Immunogenicity ... 55
7.2.1 Humoral Immune response ... 55
7.2.2 Quality of the humoral Immune response: ... 55
7.2.3 Recognition of native antigens of *P. falciparum* ... 55
7.2.4 Antigen-specific memory B-cells ... 56
7.3 Exploratory Evaluation Criteria: Immunogenicity ... 56
7.3.1 Vaccine-specific Cellular Immune Response ... 56
7.3.2 Evolution of Vaccine-specific Humoral Immune Response ... 57
8 STATISTICAL METHODS AND DATA ANALYSIS 58
8.1 Principal Objective ... 58
8.2 Determination of the Sample Size ... 58
8.3 Data Set to be analysed ... 58
8.3.1 Definition of Population ... 58
8.4 Population used in analyses ... 59
8.5 Specific analyses ... 59
8.6 Statistical Methods ... 59
8.6.1 Primary Criterion ... 59
8.6.2 Secondary Criteria ... 59
8.7 Data Management ... 60
8.7.1 Plans for analysis ... 60
8.7.2 Source documents and access ... 60
8.7.3 Data ownership ... 60
9 SITE MONITORING PLAN 61
10 QUALITY CONTROL AND QUALITY ASSURANCE 62
10.1 QA/QC Policy ... 62
10.2 Modification/Amendment of the Protocol ... 62

10.3 Archiving 62
11 ETHICS/PROTECTION OF HUMAN PARTICIPANTS 64
11.1 Ethical Standard 64
11.2 Informed Consent Process 64
11.3 Screening and study informed consent 64
11.4 Participant Confidentiality 65
11.5 Future Use of Stored Specimens 66
11.6 Publication Policy 66

List of Abbreviations

ADCI	Antibody Dependent Cellular Inhibition
AE	Adverse Events
AMANET	African Malaria Network Trust
ANOVA	Analysis of Variance
ASH	Albert Schweitzer Hospital
AS02	Adjuvant mixture of oil-in-water and monophosphoryl lipid A plus saponin
BCG	Bacille Calmette Guerin vaccine for tuberculosis
CBA	Cytometric Bead Array
CFA/IFA	Complete / Incomplete Freund's Adjuvant
CIOMS	Council of the International Organisation of Medical Sciences
cm	Centimetre
Da	Daltons
DSMB	Data Safety and Monitoring Board
EIR	Entomological Inoculation Rate
ELISA	Enzyme-linked Immunosorbent Assay
EMVI	The European Malaria Vaccine Initiative
EPI	Expanded Program on Immunization
FBC	Full Blood Count
GIM	Growth Inhibition in presence of Monocytes
GLURP-MSP3	Fusion protein derived from *P. falciparum* Glutamate-rich protein (GLURP) genetically coupled to *P. falciparum* Merozoite surface protein 3 (MSP3)
GMP	Good Manufacturing Practice
GMZ2	Product code name for GLURP-MSP3
GRAS	Generally Recognised As Safe
Hb	Haemoglobulin
hr	Hour
IFA	Immunofluorescence assay
IFAT	Immunofluorescence Antibody Test
IFNγ	Interferon γ
IgG	Immunoglobulin Gamma
iv	intravenous(ly)
kDa	kilodaltons
kg	kilogram(s)
LSP	Long Synthetic Peptides
mg	Mlligram(s)
min	Minute(s)
mL, ml	Milliliter(s)
mm	Millimetre(s)
MRU	Medical Research Unit
PBMC	Peripheral Blood Mononuclear Cells
PHA	Passive Hemagglutination Antibody
pRBCs	Parasitised red blood cells
RBC	Red Blood Cell
SAEs	Serious Adverse Events
sc	Subcutaneous(ly)
SDS-PAGE	Sodium Dodecyl Sulphate-polyacrylamide gel Electrophoresis

SEB	Staphylococcal Enterotoxin B
SMAC	Severe Malaria in African Children
SPF	Specific Pathogen Free
°C	Degrees celsius or centigrade
μg	Micrograms
μL	Microliter
vas	Visual analogue scale
WFI:	Water For Injection

PROTOCOL SYNOPSIS FOR PHASE Ib PAEDIATRIC STUDY

**Title**	A phase I, randomized, controlled, double-blind, single centre trial to evaluate the safety and immunogenicity of 30 and 100 µg of GMZ2 in Gabonese children aged 1-5 years.
**Phase**	Ib
**Population**	30 healthy children aged 1-5 years residing in Lambaréné (Gabon)
**Number of Sites**	One
**Study Duration**	16 months
**Participation Duration**	13 months
**Planned Study Period**	August 2008- November 2009
**Primary objective**	**To evaluate the safety and reactogenicity** of three doses of 30 and 100µg GMZ2, adsorbed on aluminium hydroxide, in comparison with three doses of the control vaccine (rabies), in healthy Gabonese children aged 1-5 years.
**Secondary objectives**	**To assess the humoral immune response** to the vaccine antigens GMZ2, GLURP and MSP3 by measuring the total IgG concentration and IgG isotypes against GMZ2 by ELISA.<br>**To assess B-cell memory** by memory B-cell ELISPOT.
**Exploratory Objectives**	**To assess the functionality of the immune response** by measuring the Growth Inhibition of *P. falciparum* in the presence or absence of Monocytes, and by measuring the recognition of native antigen of *P. falciparum* by IFA.
**Study Design**	Phase Ib double-blind, randomised, and controlled trial with three groups at one study site
**Randomization allocation concealment**	The Statistician will randomise and prepare sealed allocation envelopes based on the screened list of eligible children. Allocation list will be maintained by the Pharmacist and the Local Safety Monitor (LSM) in case of need to unblind the study for safety reasons.
**Estimated Time to Complete Enrolment**	Two weeks
**Trial Centre**	Medical Research Unit, Albert Schweitzer Hospital
**Description of Agent or Intervention**	Lyophilized recombinant *Lactococcus lactis* Hybrid GMZ2 [GLURP+MSP3]
**Dose**	30 & 100µg
**Route**	Intramuscular
**Control Product**	Rabies vaccine
**Vaccination Schedule**	Days 0, 28 & 56
**Follow-up duration**	One year after the first vaccination

**Serology Schedule**	D0, D28, D56, D84, and D365
**Primary Evaluation Criteria**	The safety profile will be assessed on the following criteria:<br>- Immediate reactogenicity (reactions within 30 minutes after each injection, with emphasis on allergic reaction).<br>- Local and systemic reactogenicity measured from Day 0 to Day 14 after each dose.<br>- Any adverse event resulting in a visit to a physician between each injection and one month after the third dose.<br>- Any Serious Adverse Event (SAE) occurring from inclusion and through out the study. The relationship of the adverse event to the study vaccine will be established by the investigator, using the following definitions : related, or not related<br>- Biological safety: one month after each vaccination, in reference with the baseline before the first dose, by measuring the following:<br>▪ RBC, haemoglobin, haematocrit, platelets, and WBC.<br>▪ ASAT, ALAT, total bilirubin, alkaline phosphatase, creatinine.
**Secondary Evaluation Criteria**	**The humoral response** to the vaccine antigens will be assessed by measuring the level of<br>a) Total IgG and IgG isotypes on Days 0, 28, 56, 84, and 365.<br>b) Antigen specific memory B-cell by ELISPOT on Days 0, 84, and 365
**Statistical Methods**	95% confidence intervals will be calculated for incidence of adverse events and for immune responses within each group. The analysis shall be primarily descriptive.

Study Flow Chart

Visit Number	V1	V2	V3	V4	V5	Field worker visits -[D 2, 4, 5, and 6]	V6	V7	V8	V9	V10	Field worker visits - [D 30, 32, 33, and 34]	V11	V12	V13	V14	V15	V16	Field worker visits- [D 58, 60, 61,and 64]	V17	Field worker visits [D 140, 224, and 308]	V18
Trial Timelines (Days,)	D-14	D0	D1	D3	D7		D14	D28	D29	D31	D35		D42	D56	D57	D59	D63	D70		D84		D365
Time Windows (Days or Hours)	[± 3D]		[± 6 hrs]	[± 1D]	[± 1D]		[± 2 D]	[± 3 D]	[± 6hrs]	[± 1D]	[± 1D]		[± 2 D]	[± 3 D]	[±6hrs]	[±1D]	[±1D]	[± 2D]		[± 7 D]		[± 14 D]
Visit Intervals		V1 +14 D	V2 + 24 hrs	V2+ 3D]	V2+ 7D]		V2 +2 wks	V2 +4 wks	V7+1D]	V7+ 3D	V7+7D		V7+2 wks	V7 + 4 wks	V12+ 1D	V12+3D	V12+ 7D	V12+ 2wks		V12 +4 wks		V2 +52 wks
Vaccination Doses		Vac1						Vac2						Vac3								
Eligibility Criteria	X	X																				
Inclusion & Exclusion Criteria	X	X						X						X								
Informed Consent	X	X																				
Medical History	X																					
Physical Examination	X	X	X	X	X		X	X	X	X	X		X	X	X	X	X	X		X		X
Contra-Indications Review		X						X						X								
Immediate surveillance (30 min)		X						X						X								
Solicited Reactions (1- 14Days)			X	X	X		X	X	X	X	X		X	X	X	X	X	X		X		
Unsolicited Events/Reactions		X	X	X	X		X	X	X	X	X		X	X	X	X	X	X		X		
Serious Adverse Events		X	X	X	X		X	X	X	X	X		X	X	X	X	X	X		X		X
Concomitant therapy		X						X						X						X		
Blood Sampling 7.5mL)- FBC/Chem	BS1							BS2						BS3						BS4		BS5
GMZ2 IgG, IgG isotypes		X						X						X						X		X
ELISPOT		X																		X		X
Blood smear	X																					
Field worker visit card						x						x							X		X	
Trial phase	Screening	Vaccination																			Follow-up	
Termination Record / Interim																				X		
Termination Record / Final																						X

1 BACKGROUND INFORMATION AND SCIENTIFIC RATIONALE

1.1 Background Information

1.1.1 Disease burden

Africa bears the heaviest burden of malaria. The most dangerous parasite species, *P. falciparum*, is responsible for more than one million deaths worldwide each year. More than 90% of these deaths occur among sub-Saharan African children under five years old. In areas of stable malaria transmission, 25% of all-cause mortality in children aged five years or less has been directly attributed to malaria[1]. Evidence from impregnated bed net trials in West Africa indicates that malaria could account directly and indirectly for as much as 60% of all-cause mortality in children aged less than five years old[2-4].

1.1.2 Malaria in Lambaréné (Gabon)

Malaria remains one of the leading public health problems in Gabon. The major vectors for malaria transmission are *Anopheles gambiae* and *A. moucheti* and the Entomological Inoculation Rate (EIR) in Lambaréné and its surroundings is about 50 infective bites per person per year (Sylla et al. 2000). There is little seasonal variability in transmission rates or parasite prevalence[5]. *Plasmodium falciparum* is the predominant species and responsible for 95% of all infections. *P. falciparum* parasitaemia with clinical symptoms is infrequent in adults, and occur almost solely in school and pre-school children. The level of chloroquine resistance is high, nearly reaching 100 % both *in vitro* and *in vivo*[6]. Recent data obtained from the Severe Malaria in African Children (SMAC) studies show that there is a fairly constant annual number of children admitted to the ward with *P. falciparum*, averaging about 500 children per year[7]. On average, children aged 2-12 years experience about 1.5 malarial attacks per year, with a large variability among individuals[8]. Anaemia is the most common complication, affecting about 70% of all malaria cases and, 17% of all hospitalised malaria cases have anaemia (Hb < 9 g/dL) or severe anaemia (Hb < 5 g/dL), respectively[9].

1.2 Global Epidemiology

Malaria affects 40% of the world's population with over one million deaths annually. This represents a tremendous human suffering and a burden that prevents the development of the affected endemic communities. Malaria is now almost confined to the poorest tropical areas of Africa, Asia and Latin America, but transmission is being reintroduced to areas where it had previously been eradicated. Malaria is one of the world’s greatest public health problems.

It has been estimated that the incidence of malaria in the world may be in the order of 300 million clinical cases each year. Countries in tropical Africa account for more than 90% of these cases[10] (WHO, 1999). Malaria mortality is estimated at almost one million deaths worldwide per year. The vast number of malaria deaths occurs among young children in Africa, especially in remote rural areas with poor access to health services. Other high risk groups include women during pregnancy, and non-immune travellers, refugees, displaced persons, or labour forces entering into endemic areas. Malaria infection has been increasing over recent years due to a combination of factors including increasing resistance of malarial parasites to chemotherapy

and increasing insecticide resistance of the Anopheles mosquito vector, ecological and climate changes, increased international travel to malaria-endemic areas.

1.3 Prevention and Control of Infection among Humans

The disease burden persists for several reasons. Among them, is the failure to achieve adequate coverage with, and reduced pricing of existing tools such as drugs and insecticide-treated bed nets. There is user ignorance of how to use these tools. The decreasing effectiveness of existing tools (e.g. emergence of drug resistance, particularly to chloroquine and sulfadoxine-pyrimethamine, and, in south-east Asia, resistance to third- and fourth-line anti-malarials; resistance to insecticides, including pyrethroides) is a major challenge. The increasing emergence of insecticide resistant vectors and drug resistant parasites calls for investment in new and better control tools[10] (WHO, 1994).
Malaria vaccines hold the potential to dramatically alleviate the burden of malaria. However, our understanding of the mechanisms underlying protective immunity is incomplete and specific markers of protection still need to be defined.

1.4 Malaria vaccines

An effective malaria vaccine will require the induction of appropriate humoral and cellular immune responses against several key parasite antigens expressed during the various stages of the parasite life cycle. Each stage in the life cycle provides an opportunity for a vaccine. An asexual stage blood stage vaccine will induce immunity that controls parasitaemia and therefore prevent clinical complications increasing the risk of death.
Two lines of evidence suggest that a malaria vaccine is attainable.
Firstly, it is a well-established observation that repeated exposure to malaria parasites can lead to the development of solid clinical immunity, a status of premonition with concomitant existence of parasites and protective antibodies. Clinically immune individuals generally have a lower parasite density and the immunity is quite effective at reducing mortality.
Secondly, experiments in animal models as well as in humans have established that immunisations can induce immunity against subsequent challenge with parasites suggesting that vaccination can become a realistic tool for malaria control.
In now classical experiments, Cohen and colleagues demonstrated that the passive transfer of antibodies, purified from clinically immune individuals, could ameliorate acute malaria attacks in African children with life-threatening *P. falciparum* infections[11] (Cohen, 1961). Druilhe and coworkers confirmed Cohen’s results[12] (Druilhe, 1991). They showed that IgG from clinically malaria-immune West Africans were able - in a strain-independent manner - to substantially decrease the parasite load in asymptomatic Thai children with drug resistant *P. falciparum* malaria[12].
These groundbreaking passive transfer experiments have proven that antibodies are crucial in reducing /eliminating the asexual stage parasite load.
In vitro investigations with the same “protective” IgG preparations (Druilhe, 1991) demonstrated that antibodies, on their own, do not substantially inhibit parasite growth, but act synergistically with blood mononuclear cells to control parasite multiplication[13] (Bouharoun-Tayoun, 1992). This parasite containing mechanism is referred to as “antibody-dependent cellular inhibition” (ADCI) (Bouharoun-Tayoun, 1995; Khusmith, 1983; Lunel, 1989). Recent studies have demonstrated that binding of cytophilic antibodies, such as IgG1 and IgG3, to blood mononuclear cells via their FcγIIa receptors trigger the release of killing factors such as tumor necrosis factor-α[14, 15, 16] (Bouharoun-Tayoun, 1990).

Immuno-epidemiological studies support the in vivo relevance of a monocyte-dependent, antibody-mediated mechanism by showing a correlation between the acquisition of clinical immunity and levels of IgG1 and IgG3 antibodies, which bind well to the monocyte FcγRIIa receptor[17] (Aribot, 1996; Sabchareon, 1991). The putative involvement of this receptor in the development of immunity against clinical malaria is also supported by the finding that allelic polymorphism in FcγRIIa is associated with differential susceptibility to *P. falciparum* malaria (Shi, 2001). Kenyan infants homozygous for the FcγRIIa-Arg131 allele are reported to be less at risk from high-density *P. falciparum* infections compared with children with the heterozygous Arg/His131 genotype. Since the FcγIIa-Arg131 genotype (but not the FcγIIa-His131 genotype) binds strongly to IgG1 and IgG3, this finding supports the notion that monocyte-mediated killing of *P. falciparum* is an important mechanism for parasite containment in vivo[18]. Additionally, Aucan et al found that levels of specific IgG2 antibodies - but not IgG3 and IgG1 - were associated with protection from clinical malaria in a population from Burkina Faso[19] (Aucan, 2000). Subsequent sequencing of FcγRIIa revealed that 70% of the study participants had the FcγRIIa-H131 allele. This allele binds strongly to IgG2, suggesting that IgG2 is acting as a cytophilic subclass in this population[20] (Warmerdam, 1991). Collectively, these observations suggest that the FcγRIIa genotype is an important factor for the development of immunity to clinical malaria and lends support to the validity of the in vitro ADCI model.
These observations support the development of a multi-component vaccine containing *P. falciparum* antigens, which have been identified on the basis of the above and have been tested individually in clinical phase I trials.

1.4.1 GLURP antigen

The identification and subsequent cloning of the gene for the *P. falciparum* Glutamate-rich protein (GLURP) stems from the work of Jepsen and co-workers on soluble antigens, the so-called exoantigens[21] (Theisen, 2002, 2000, 1998). These antigens are present in the parasitophorous vacuole of parasitized red blood cells. They become associated with the surface of the merozoite at the time of schizont rupture and they are also present in soluble form in the supernatants of in vitro cultures of *P. falciparum* as well as in the plasma of patients with *P. falciparum* malaria[22] (Jepsen, 1980; Wilson, 1969). The importance of these antigens is suggested by the observations that affinity-purified human antibodies to exoantigens can inhibit the growth of *P. falciparum* in vitro (Jepsen, 1983) and that exoantigens can induce protective immunity against an experimental *P. falciparum* infection in *Saimiri sciureus* and *Aotus nancymai* monkeys[23, 24] (James, 1991, 1985). The potential protective effect of the exoantigens is further suggested by immuno-epidemiological studies showing that levels of cytophilic subclasses are associated with protection against high levels of parasitemia (Luty, 1994) and with protection against clinical disease[25,26] (Chumpitazi, 1996). The reverse relationship was found for the levels of non-cytophilic IgM and IgG2 subclasses (Chumpitazi, 1996; Luty, 1994) leading to the hypothesis that development of anti-disease immunity in young children depends on IgM antibodies against exoantigens, whereas the progressive development of antiparasite immunity in older children would be reflected by the switch to exoantigen-specific cytophilic subclasses [25](Luty, 1994). Finally, it has been proposed that vaccination with the exoantigens is a possible strategy for prevention of disease symptoms in malaria[27] (Playfair, 1990).

1.4.2 MSP3 antigen

The ADCI of parasite growth assay was used as a means of selecting molecules capable of inducing protective immunity to malaria. An antibody specificity, which promotes parasite killing mediated by monocytes, was identified in the sera of clinically protected participants. This

antibody was directed against a novel Merozoite Surface Protein (MSP3) of a molecular mass of 48 kDa[28] (Oeuvray, 1994). Purified IgG from protected participants are effective in ADCI and those directed against MSP3 are predominantly cytophilic. In contrast, in non-protected individuals, whose antibodies are not effective in ADCI, anti-MSP3 antibodies are mostly non-cytophilic[29]. A region in MSP3, targeted by antibodies effective in the ADCI assay, was identified and its sequence determined (MSP3b); it contains an epitope, which is not defined by a repetitive structure, and does not appear to be polymorphic. Antibodies raised in mice against a peptide containing this epitope, as well as human antibodies immunopurified on this peptide, elicit a strong inhibition of *P. falciparum* growth in ADCI assay, whilst control antibodies, directed to peptides from other molecules, do not. The correlation between isotypes of antibodies produced against the 48 kDa epitopes, clinical protection, and ability of specific anti-MSP3 antibodies to block the parasite schizogony in the ADCI assay suggested that this molecule is involved in eliciting protective mechanisms[30]. Moreover, in contrast with other vaccine candidates, the target B and T-cell epitopes were found to be fully conserved in 67 *P. falciparum* isolates[13] (Bouharoun-Tayoun, 1992).

Immuno-epidemiological studies: Using a study design incorporating temporally close clinical follow-up, clinical data were compared with the immune responses to five malaria vaccine candidate antigens including MSP3. The clinical-epidemiological design set up in the village of Dielmo (Senegal) provides a very close monitoring of malaria, as it includes daily follow-up, 24 hours a day, seven days a week, of the 247 inhabitants on a year-round basis. Since clinical protection was previously shown to be dependent on the cytophilic subclasses of immunoglobulins, the isotype-specific distribution of antibodies to the blood-stage antigens AMA1, MSP1, MSP2, MSP3 and RESA, was determined by standardised ELISA methods[31] (Aribot, 1996).
Results show that, for each of the two years of follow-up and for each age group, there is a strong correlation between protection against malaria attacks and the ratio of cytophilic (IgG1 and IgG3) to non-cytophilic (IgG2, IgG4 and IgM) antibodies against the MSP3 antigen. At the level of individuals, the presence of IgG3 against MSP3 was strongly predictive of the clinical outcome in each age group, and was independent of age. This predictive value was not found for other isotypes or for antibodies directed to any of the four other malarial antigens that were studied in parallel. The results suggest that antibodies to a small non-polymorphic region of a single well-defined antigen, MSP3, can determine to a large extent the status of resistance to clinical malaria. Hence, IgG3 production against this dominant epitope appears as a valuable marker of protection against malaria. More generally, the present study indicates that ADCI may be used as a potent marker to select among numerous malarial antigens those, which are targeted by clinically protective immune responses. Since protective responses against the MSP3 were demonstrated in individuals of all ages, including young children, the data also suggest that these responses can be acquired by young children, and may therefore be induced by vaccination in individuals of all ages[32].

1.5 Name and description of the investigational products

GMZ2 (GLURP-MSP3 bivalent vaccine) is a recombinant hybrid protein between GLURP and MSP3 expressed in the *L. lactis* expression system, which is suitable for GMP production and testing in human volunteers.
This production host is superior to existing prokaryotic hosts such as *E. coli*, because it allows efficient secretion of the recombinant protein into the culture supernatant and because *L. lactis* is a Gram-positive organism, which does not produce endotoxins.

1.6 Summary of findings from non-clinical studies

Pre-clinical experiments have further demonstrated that the GLURP-MSP3 hybrid vaccine is superior to a vaccine containing a mixture of the individual molecules because the latter tends to elicit an immune response against B-cell epitopes in either GLURP or MSP3 (see below).

1.6.1 Antigenicity

The antigenicity of the GLURP-MSP3 hybrid vaccine has been evaluated by ELISA against IgG antibodies from 71 adults Liberians clinically immune to malaria. Serial dilutions of all plasma samples were tested on separate plates coated with either the GLURP-MSP3 hybrid vaccine or the individual GLURP and MSP3 fragments and the antigen-specific titre was determined as the dilution giving an absorbance of 1.00. As expected, different plasma contained different amounts of GLURP and MSP3-specific IgG antibodies. In general, GLURP-MSP3 hybrid-specific antibody titres exceeded those recorded with the individual GLURP and MSP3 antigens suggesting that the GLURP-MSP3 hybrid molecule provides an adequate presentation of GLURP and MSP3 antigenic determinants, respectively[33].

1.6.2 Immunogenicity

To determine whether the GLURP-MSP3 hybrid molecule is a superior immunogen compared to a mixture of the individual $GLURP_{25\text{-}514}$ and $MSP3_{212\text{-}380}$ molecules, groups of BALBc/CF1 mice were each immunized subcutaneously with the hybrid molecule in Montanide or with the individual $GLURP_{25\text{-}514}$ and $MSP3_{212\text{-}380}$ proteins combined in either one syringe or injected separately at two different sites. Sera collected 35 days after the first injection, were tested for IgG antibody reactivity against GLURP and MSP3, respectively. While the mean GLURP-ELISA titre is only marginally higher in the hybrid group than in the other two groups, mean MSP3-ELISA titre is 4.3-fold higher[34] (Kruskal-Wallis test, $P<0.004$) in the group receiving the hybrid compared to the group receiving both $MSP3_{212\text{-}380}$ and $GLURP_{25\text{-}514}$ at two different sites. At the individual level, mice immunized with the hybrid reacted strongly with both GLURP and MSP3 domains whereas mice immunized with a combination of two molecules tended to mount a predominant antibody response against either GLURP or MSP3. The anti-hybrid IgG antibodies are mainly directed against the GLURP-derived P3, P4, P11, and S3 peptides containing known epitopes for human antibodies; however peptides P5 and P9, which do not contain such epitopes, were also recognized. Whereas the GLURP and MSP3-specific IgG subclass profiles are similar for all vaccine formulations, GLURP-specific IgG antibodies use preferentially the Kappa light chain and MSP3-specific IgG antibodies preferentially the Lambda light chain. This difference in light chain was found for all GLURP or MSP3-specific antibodies whether raised against the hybrid or the mixtures of the individual molecules[34].

The specificity of mouse antibodies to the hybrid was also analyzed by competition-ELISA. It appears that antibodies to the hybrid are purely GLURP and MSP3-specific, since a mixture of soluble $GLURP_{25\text{-}514}$ and $MSP3_{212\text{-}380}$ could completely inhibit the binding of anti-hybrid antibodies to immobilized $GLURP_{27\text{-}500}$-$MSP3_{212\text{-}380}$. Thus, the construction of a GLURP-MSP3 hybrid molecule has not led to the creation of new B-cell epitopes in the overlapping area.

The immunogenicity of the recombinant GLURP and MSP3 was also investigated by immunoblotting of parasite-derived proteins with sera from mice immunized with each of the three recombinant proteins, hybrid, $GLURP_{25\text{-}514}$ and $MSP3_{212\text{-}380}$, respectively. Plasma from mice immunized with $GLURP_{25\text{-}514}$, $MSP3_{212\text{-}380}$, and the hybrid recognized polypeptides of approximately 220,000 Da, 48,000 Da, and both, the apparent molecular masses previously found for GLURP and MSP3 by SDS-PAGE, respectively[34].

1.6.3 Safety and immunogenicity in Saimiri monkeys

The safety of the GLURP-MSP3 hybrid protein has been tested in 18 *Saimiri sciureus* monkeys. Fifty µg of the GLURP-MSP3 hybrid protein was thoroughly mixed with three different adjuvants: alum (Superfos Biosector, Denmark), Montanide ISA720 (Seppic, France) and Freund's (Sigma, USA) (final volume was 500µl) and administered subcutaneously at four different points on the back of the animals (six animals per adjuvant). Three doses were administered to each animal, on Days 0, 30 and 90. In the case of Freund's, the animals received the complete (CFA) adjuvant in the first dose and the incomplete (IFA) in the other two. Blood was collected on Days 0, 30, 60, 90 and 135 for immunological assays. In each of these manipulations, haematological parameters were evaluated, monkeys were weighed and the sites of injection were examined to check for local adverse reactions. Local reactions were not observed in the 12 monkeys immunized with the GLURP-MSP3 hybrid protein in alum or Montanide ISA720[35]. Following immunisations, no major changes in weight or haematological parameters were observed. Monkeys that received the CFA/IFA (six animals) developed local inflammatory reaction with swelling at the injection site due to the adjuvant. Reactions were observed mainly after the second injection, and increased in size and severity with the third dose.

1.7 Summary of previous clinical trials

The synopsis of the previous clinical trials involving the components of this hybrid is provided hereunder.

1.7.1 Phase Ia trial of MSP3-LSP in "naïve" European adults:

A first phase I trial was conducted in Switzerland, on healthy adults, who had not been exposed to malaria. The MSP3-LSP vaccine was given to 35 persons, in three subcutaneous injections. Primary-vaccination consisted of two doses given at a one-month interval. The booster dose was given three months after primary-vaccination. Four different doses: 10µg, 20µg, 30µg or 100µg and two adjuvants, Montanide 720 and aluminium hydroxide, were studied.
The vaccine showed a very good systemic tolerance, whatever the adjuvant or dose, with no systemic reactions observed during the study. In contrast, local tolerance was clearly linked to dose, adjuvant and injection number. The functionality of the immune response, as measured by the ADCI assay, showed that antibodies induced by vaccination could lead to an inhibition of *P. falciparum.*
To conclude, at a dose of 30µg, the MSP3 vaccine selected as best-tolerated by healthy adults not exposed to malaria, and led to the production of antibodies capable, in vitro, of neutralizing the *P. falciparum* parasite (Druilhe, 2002).

1.7.2 Phase Ib trial of MSP3-LSP in exposed Burkinabé male adults

The second trial with MSP3 was a phase Ib trial conducted in Burkina Faso among semi-immune adults. A total of 30 participants enrolled and all of them received three vaccine doses at 30µg or tetanus toxoid in the same schedule of 0, 1 and 4 months. All participants completed the study *per protocol*.

In summary, both vaccines were well tolerated; no unexpected adverse reactions were reported. No serious adverse events were observed. Not surprisingly, the humoral immune response induced by the MSP3-LSP vaccine in these semi-immune adults was much less than that induced in the malaria naïve Swiss adults. The response in the MSP3-LSP vaccine group to

stimulation with MSP3-LSP antigen increased after vaccination, while that in the tetanus group appeared unchanged.
The pattern of IFN gamma responses to stimulation with Passive Hemagglutination Antibody (PHA) antigen was very similar in the two groups with a slight decrease between Day 0 and Day 56. IFN gamma responses to stimulation with tetanus toxoid antigen were unchanged post vaccination. IFN gamma responses to stimulation with MSP3-LSP antigen appeared to remain stable in the MSP3-LSP group after vaccination, while in the tetanus group, they showed a similar decline to that observed with PHA[56] (Sodiomon B Sirima, 2006).

1.7.3 Phase Ia trial of GLURP in "naïve" European adults

A phase I clinical trial was conducted in healthy adult volunteers applying the long synthetic peptide GLURP85-213 combined with either aluminium hydroxide (alum) (n=18) or Montanide ISA 720 (Mon) (n=18). The vaccine (10, 30 or 100µg) was administered subcutaneously at days 0, 30, and 120.
The vaccine induced dose-dependent cellular and humoral immune responses, with high levels of *in vitro* IFNγ and (cytophilic) antibodies that recognize parasites in IFA. Plasma samples collected 30 days after the last immunization induced in presence of monocytes inhibition of parasite growth *in vitro*. The inhibition was dose-dependent and correlated significantly to the plasma IFA titre against *P. falciparum* parasites.

In conclusion, two injections of 30 µg GLURP85-213 LSP with Montanide ISA720 or three injections of 30 µg GLURP85-213 LSP with alum hydroxide are safe and immunogenic, generating a strong IgG1/IgG3 *P. falciparum*-specific response of biologically active antibodies *in vitro*.

1.7.4 GMZ2 (GLURP-MSP3) Phase I trials

1.7.4.1 Phase Ia results from "naïve" German adults

The phase Ia trial of GMZ2 vaccine (GLURP + MSP3 hybrid) in Germany involved the administration of 10, 30 and 100µg of GMZ2 malaria vaccine given with Aluminium Hydroxide (Alhydrogel®) as adjuvant. Thirty volunteers in three groups of 10 were randomised to receive 10, 30 or 100µg of GMZ2 subcutaneously administered four weeks apart in a dose-escalating design. Volunteers were followed up for 14 days after each vaccination to monitor adverse events. Blood samples for immunological assays were taken at baseline and 28 days after each vaccination.
All volunteers were seen on Day 84 (four weeks post third dose) and were included in the intention to treat interim analysis. Twenty-nine out of thirty volunteers received all three doses of the vaccine. One volunteer was excluded for reasons unrelated to GMZ2 vaccinations. All vaccinations were well tolerated and no Serious Adverse Events were observed. All local and systemic adverse events were classified as mild or moderate. Most common solicited adverse events were local reactions at the injection site, fatigue and headache. There were no substantial changes in haematological and clinical chemistry results. Preliminary conclusion was that at all the three different doses used in the trial, the GMZ2 vaccines were safe and well tolerated.
Immunogenicity analysis, total IgG against GLURP, MSP3 and the combined vaccine antigen (GMZ2) was determined by ELISA. At all vaccine dose levels (10, 30 & 100µg), there was a significant increase of IgG against all antigens four weeks after the second and third doses. One week after the first vaccination GMZ2 responses were significantly higher in the 100 µg

compared to the 10 µg group (p=0.003). All other group comparisons yielded no significant difference. On Day 84 vaccine induced IgG against the vaccine antigen (as well as the individual GLURP and MSP3 domains) reached levels comparable to randomly chosen, adult, semi-immune individuals from Lambaréné (median $_{\text{Lambaréné}}$ = 0.9 mg/dl, median $_{\text{10 ug GMZ2}}$ = 1.2 mg/dl, median $_{\text{30 ug GMZ2}}$ = 1.4 mg/dl, median $_{\text{100 ug GMZ2}}$ = 1.4 mg/dl).
In conclusion, it was noted that the safety and immunogenicity profile was similar at all vaccine dose levels tested. Thus, using the highest possible safe dose (100µg GMZ2) in the Phase Ib trial in semi-immune African adults in Gabon will allow gradual reduction in dose levels when age de-escalation trials commence in children and toddlers in Africa.

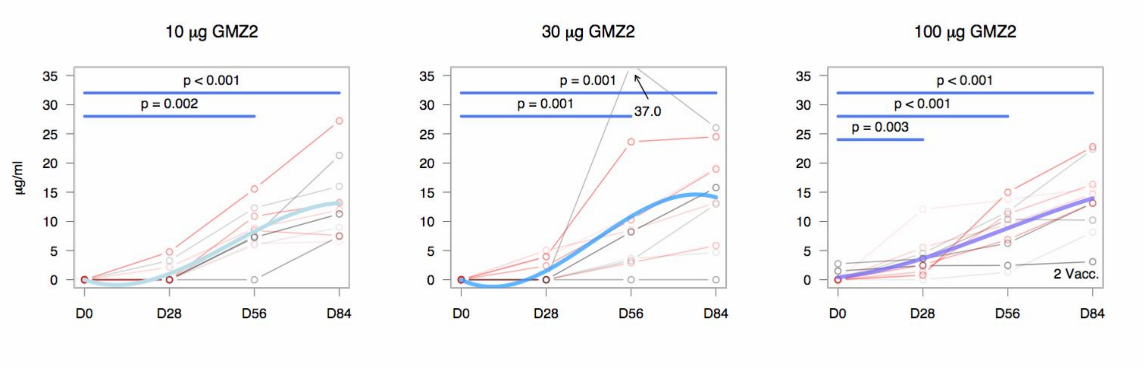

Figure 2: *Total IgG against GMZ2. Levels on the y-axis are in µg/ml. All p-values were calculated using non-parametric methods (Kruskal-Wallis χ2). Note the significant difference between the 10 and 100 µg groups on D28. D0: baseline values; D28: 4 weeks after first vaccination; D56: 4 weeks after second vaccination; D84: 4 weeks after third vaccination;*

1.7.4.2 Phase Ib interim results from Gabonese malaria exposed adults

The phase Ib trial of GMZ2 vaccine is currently ongoing at Lambaréné Gabon. It is a randomised, controlled trial, double-blind trial evaluating the safety and Immunogenicity of 100µg GMZ2 administered subcutaneously versus rabies vaccine at a schedule of 0, 28, and 56 days in healthy Gabonese males aged 18-45 years.
This study enrolled 40 participants in two arms, and volunteers were actively followed up for 14 days after each vaccination to monitor adverse events. Blood samples for immunological assays were taken at baseline and 28 days after each vaccination. All volunteers were seen on Day 84 (four weeks post third dose) and were included in the intention to treat interim analysis. Thirty nine volunteers received all three doses of the scheduled vaccinations. One volunteer was excluded from receiving the third dose when he was diagnosed with pulmonary tuberculosis. All vaccination were well tolerated and no Serious Adverse Events (SAEs) related to vaccines were observed. Overall, five serious SAEs not related to vaccinations have been reported and adequately followed up.
All local and systemic adverse events were monitored and only one solicited local adverse event was reported as grade 3 (severe) and it resolved within 48 hours. No grade 3 solicited systemic adverse events or grade 3 unsolicited adverse events have been observed. The most common solicited adverse events were local reactions at the injection site, fatigue diarrhoea and headache. There were no substantial changes in the biological parameters measured except for one case reported as SAE who had high eosinophilia which is still under investigation, but it had no reported clinical consequences.

Table 1: Summary of solicited local adverse events (maximum observed grade)

	Vaccination 1				Vaccination 2				Vaccination 3			
AE	Grade/ Total	1	2	3	Grade/ Total	1	2	3	Grade/ Total	1	2	3
Site Pain	14	14	0	0	30	30	0	0	17	17	0	0
Erythema	0	0	0	0	0	0	0	0	0	0	0	0
Induration	2	2	0	0	4	4	0	0	2	2	0	0
Oedema	2	2	0	0	0	0	0	0	0	0	0	0
Pruritis	1	1	0	0	1	1	0	1	1	1	0	0
Local heat	1	1	0	0	0	0	0	0	0	0	0	0
Total	20	20	0	0	35	35	0	1	20	20	0	0

Based on these interim safety results, the clinical development team recommended that GMZ2 is safe in malaria exposed adults and should progress to children studies for evaluation of safety and immunogenicity. The immunological results from this study are expected to be available within the first quarter of 2008.

1.8 Rationale

1.8.1 Risks and benefit to human participants

The sterility of the product and the absence of toxicological effects are guaranteed by the results of the quality controls performed by Henogen and the pharmaco-toxicological study performed by Scantox. Therefore, we are not aware of any factor that should dramatically alter the safety profile of the active ingredients from what has so far been observed.

1.8.2 Rationale for adjuvant selection

Aluminium hydroxide, is most widely used and generally accepted for use in human vaccines. Results in other malaria vaccine trials show that good immune responses can be obtained. Moreover, in the phase Ia trial, it has emerged as the best adjuvant among two others that were compared i.e. Montanide and AS02.

1.8.3 Rationale for dosage selection

Studies with either MSP3 or GLURP as Long Synthetic Peptides indicate that both peptides induce a dose-dependent immunological response. This study intends to evaluate previously used 100µg of GMZ2 as well 30µg of GMZ2 as this is the first time the product will be tested in children. This approach will allow for identification of any dose dependant toxicity of the product and also determination of the safest dose of the vaccine that is immunogenic in a population close to the targeted Expanded Program of Immunization (EPI) age group.

1.8.4 Justification for 0, 28, 56 Day Schedule

In this adult trial, we seek to evaluate the safety as has been demonstrated in the adult phase Ia trial in Europe and phase Ib trial in Gabon. The target group for this vaccine is the infant population to be vaccinated through the EPI, which includes immunization of infants at four week-intervals starting at the age of six weeks for oral poliomyelitis vaccine and DTP

(Diphtheria-Pertussis-Tetanus) in a 0,1, 2 months order. It is envisioned that ultimately, the EPI would be the only efficient means of delivering a vaccine against malaria in resource constrained areas; therefore, all trials of this product will be done using the 0, 28, 56 schedule.

1.8.5 Overview of the GMZ 2 clinical development plan

The clinical development of the candidate malaria vaccine GMZ2 is to progress in following distinct steps;

1) Phase Ia dose-finding and adjuvant-selection trial in malaria naïve adults (at Tübingen, Gemany);

2) Phase Ib trial in malaria-experienced adults (currently ongoing study);

3) Phase Ib study in Gabonese children aged 1-5 years;

4) Phase Ib study in a few African adults with the final formulation of GMZ2;

5) Phase IIb multi-centre efficacy trial involving the children 1-5 years old; and

6) Phase III large multi-centre efficacy trial in children.

1.9 Comparator Vaccine: Rabies vaccine

1.9.1 Rationale for rabies vaccine

Having a comparator vaccine is useful in Phase I trials conducted in malaria-endemic areas, since background immunity and natural exposure to malaria may make it difficult to interpret immunogenicity data. In this setting, rising titres of antibody to GMZ2 could be due to immunization or to natural exposure or both. The use of a control group will permit comparison of immune responses and will result in a clearer interpretation of serological results. While a placebo control group would accomplish this same end, using a vaccine that is beneficial to the participants further increases the benefit to risk ratio, which is always relatively low in a Phase I trial. We have chosen to use rabies vaccine as the comparator for the following reasons: i) benefit for participants who will receive rabies vaccine because the have not received this vaccine, ii) rabies vaccine is rarely used in Gabon and in Africa, iii) rabies vaccine is a protein, which is safe and well tolerated and efficacious, iv) rabies vaccine can be administered in the 0, 1 & 2 month schedule that suits the schedule of the study vaccine.

Among human infections, rabies is believed to be the tenth most common cause of death. Once clinical symptoms have occurred, the disease is almost invariably fatal. In the majority of industrialized countries, human rabies is under control, mainly due to oral vaccination of wildlife and mandatory parenteral vaccination of domestic animals. Rabies is currently an incurable disease. Antiviral agents, interferon and massive doses of rabies immune globulin have been used to treat human cases, but seem only to prolong the clinical course without affecting fatality. However, post-exposure treatment initiated at an early stage using rabies vaccine in combination with rabies immune globulin may be 100% effective in preventing death. Given pre-emptively, modern rabies vaccines produce an antibody response in over 99% of vaccinees.

1.9.2 Safety of Rabies vaccine

Although associated with mild and transient reactions, all the cell-derived rabies vaccines are considered safe. With human diploid cell vaccines, which are most thoroughly investigated, pain, erythema and swelling or itching at the injection site occur among 30%–74% of the recipients. Systemic reactions involving headache, nausea, abdominal pain, muscle aches or dizziness are reported among 5%–40% of vaccinees, and allergic oedema in 0.1%. One study reports fever among 3.6% of recipients of the human diploid cell vaccine. Systemic allergic reactions characterized by generalized urticaria accompanied in some cases by arthralgia, angiooedema, fever, nausea and vomiting have been reported. They are uncommon in persons receiving primary vaccination, but have occurred in up to 6% of persons receiving a booster dose, with onset after 2–21 days. These reactions have been shown to follow the development of IgE antibodies to β-propiolactone altered human serum albumin in the vaccine (β-propiolactone is used as an inactivating agent).

According to the manufacturers of purified Vero cell rabies vaccine and purified chick embryo cell vaccine, allergic reactions are very rare after both primary and booster doses with these vaccines. Studies of the purified vero-cell rabies vaccine reported local and general reactions in 10.6% of post-exposure treatment patients and complaints of mild to moderate reactions in 7%. Also, among intradermal or intramuscular recipients of this vaccine, low-grade fever was the only significant systemic event, occurring in 8% of all subjects and most frequently following intramuscular vaccination. In the same study, pruritus at the injection site was the only significant local reaction. Among 88 healthy adults receiving a total of 292 doses of purified chick embryo cell vaccine, 16.4% reported local side-effects, whereas 15.1% reported general symptoms.

In this study we shall use the recommended rabies vaccine in Gabon and all vaccinees will be closely monitored.

1.10 Potential Risks and Benefits

1.10.1 Potential Risks

Risks associated with both GMZ2 and rabies vaccine vaccinations include local inflammatory reactions to the injected product, such as injection site pain and swelling. Systemic effects generally associated with vaccines may include flu-like syndrome, fever, chills, nausea/GI symptoms, headache, malaise, myalgia and arthralgia. While rare, allergic reactions, including life-threatening anaphylaxis, are associated with many vaccine preparations and must therefore be considered as a potential risk in this study. Risks associated with drawing blood include fainting, infection and bruising.

Free medical treatment will be provided to enrolled participants during the active vaccination phase and the surveillance period, at a level that meets or exceeds the local Gabonese health care standards of medical diagnosis and treatment. Medical care for illnesses not related to

vaccination will not extend beyond the study period. Medical care for illnesses related to vaccination will extend, at minimum, until the condition has resolved or stabilized.

1.10.2 Known Potential Benefits

Participants may not receive any direct benefit from the experimental vaccine in this study. However, they will receive follow-up medical care at the Albert Schweitzer Hospital. During the conduct of the study, participants randomized to receive rabies vaccine will benefit from this in the sense of the reduced risk of rabies, which is present in this region, where there is a large number of stray and domestic dogs which are not vaccinated. At the end of the study all participants will be informed of the vaccine they received. Participants randomized to the GMZ2 vaccine will be offered rabies immunization at that time. This will be done at the recommended schedule of 0, 7, 28 and 365 days.

Should this vaccine be successfully registered, the children in the Gabon and the entire sub-Saharan region endemic to malaria will benefit from the vaccine protection against malaria.

2 OBJECTIVES

2.1 Primary Objective

To evaluate the safety and reactogenicity of three doses of 30 and 100µg GMZ2, adsorbed on aluminium hydroxide, in comparison with three doses of the control vaccine (rabies), in healthy Gabonese children aged 1-5 years. This will be done through evaluation of the following:

1. Immediate reactogenicity; defined as any systemic adverse reactions occurring within 30 minutes after each injection,
2. Local and systemic reactogenicity measured from Day 0 to Day 14 after each dose,
3. Any unsolicited Adverse Event resulting in a visit to a physician between each injection and one month after the third dose,
4. Any Serious Adverse Event (SAE) occurring from the inclusion throughout the study.

Biological safety, one month after each vaccination, in reference with the baseline level the following :

1. RBC, haemoglobin, haematocrit, platelets and WBCs
2. Creatinine, ASAT, ALAT, total bilirubin and alkaline phosphatase.

2.2 Secondary Objectives

2.2.1 Humoral Immune Response

To assess the humoral response to the vaccine antigens GMZ2, GLURP and MSP3 by measuring total IgG by ELISA. Anti-GMZ2 specific memory B-cell-responses and IgG-subtypes will be assessed by ELISPOT and ELISA, respectively.

1. IgG responses at Days 0, 28, 56, 84, and 365.
2. IgG subclasses at Days 0 and 84
3. Antigen-specific memory B-cells by ELISPOT at Days 0, 84, and 365

2.3 Exploratory Objectives

To assess the functionality of the immune response by measuring the Growth Inhibition of *P. falciparum* in the presence or absence of Monocytes, and by measuring the recognition of native antigen of *P. falciparum* by IFA.

3 STUDY DESIGN

This will be a randomised, controlled, double blind trial with three arms including: 10 children to receive 30µg GMZ2, 10 children to receive 100µg GMZ2 and 10 children to receive rabies vaccine.

3.1 Overview

- Randomized, controlled, double-blind phase I study
- One trial centre
- Safety oversight by a Safety Committee
- Study monitoring by AMANET
- Screening will be done within 14 days prior to the first vaccination
- Vaccination schedule will be on study days 0, 28 +/- 3, and 56+/- 3
- Route of administration will be intramuscular on the deltoid muscle, alternately starting with the left side.
- Study duration will be approximately 13 months per participant
- Each participant will attend 16 standardised visits at the trial centre (Inclusion visit D-14; D0; D1; D3; D7; D14; D28; D29; D31; D35; D42; D56; D57, D70, D84, D365).
- Blood samples will be taken by venopuncture at five time points at D-14; D0; D28; D56; D84, and D365. Biological parameters and serological will be assessed at these time points as well as serological parameters. A maximum total of 7.5 mL blood will be taken at each blood sampling. For each participant a maximum of about 37.5 mL blood will be taken during the whole study.
- Twenty four hours, 3 days, 7 days and 14 days after each vaccination a visit is planned to monitor adverse events at D1, D3, D7, ,D14, D29, D31, D35, D42, D56, D57, D 59, D63 and D70
- Follow-up of serious adverse events (SAEs) until resolution
- The primary analysis will be conducted for all primary and secondary endpoints at a data-lock-point one month post vaccination 3 (study Day 84), after which the Primary

Study Report will be produced. These results will be used to decide on the go criteria for the next trial in children.

- The study will then continue in a single-blind fashion. Data gathered during this period will be reported in an addendum report.

- At the end of the study participants will be informed which vaccine they received and those who received malaria vaccine will be offered immunization with the rabies vaccine.

3.2 Site description

The study will be conducted at the Medical Research Unit of the Albert Schweitzer Hospital in Lambaréné Gabon. Lambaréné, a town with approximately 20,000 inhabitants, is located in the Moyen Ogooué province of Gabon near the equator in the Central African rain forest. There is very little variation in temperature and humidity throughout the year, but rainfall varies with considerably less rainfall during the dry season from July to August. A population of approximately 50,000 (Lambaréné and surroundings) inhabitants are served by two hospitals in Lambaréné, with approximately 90 % of children treated in the Albert Schweitzer Hospital.

The Medical Research Unit of the Albert Schweitzer Hospital was established in 1981 and is integrated in the routine services of the Albert Schweitzer Hospital. The scientific focus is on malaria research, and main areas of interest are parasite biology, pathophysiology, pathogen-host interaction, host immune response, clinical aspects and chemotherapy of malaria. Other areas of research include schistosomiasis, filariasis and mechanisms of allergy development.

The centre has an excellent track record of clinical trials, conducted to the standard of ICH - GCP guidelines and published in high-ranking journals. The centres track record of conducting malaria vaccine trials is increasing and presently the centre is involved with GMZ2 and GSK's RTS,S malaria vaccine trials.

3.3 Participant Inclusion Criteria

- Children age 1-5 years inclusive at the time of screening;

- Residing in Lambaréné for the duration of the study;

- Written informed consent obtained before screening and study start, respectively;

- Available to participate in follow-up for the duration of study (13 months);

- General good health based on history and clinical examination.

3.4 Participant Exclusion Criteria

- Previous vaccination with any other malaria candidate vaccine.
- Concomitant vaccination with a investigational vaccine or a rabies vaccine;
- Use of a investigational or non-registered drug or vaccine other than the study vaccine(s) within 30 days preceding the first study vaccination, or planned use up to 30 days after the third vaccination;
- Chronic administration (defined as more than 14 days) of immuno-suppressants or other immune-modifying drugs within six months prior to the first vaccination. This includes any dose level of oral steroids or inhaled steroids, but not topical steroids;
- Confirmed or suspected immunosuppressive or immuno-deficient condition, including human immunodeficiency virus (HIV) infection;
- Confirmed or suspected autoimmune disease;
- History of allergic reactions or anaphylaxis to immunizations or to any of the vaccine components, or of serious allergic reactions to any substance, requiring hospitalization or emergent medical care;
- History of splenectomy;
- Laboratory evidence of liver disease (Alanine aminotransferase [ALT] greater than 1.25 times the upper limit of normal (<45 U/L) of the testing laboratory);
- Laboratory evidence of renal disease (serum creatinine greater than the upper limit of normal of the testing laboratory, or more than trace protein or blood on urine dipstick testing);
- Laboratory evidence of haematological disease (absolute leukocyte count 5.4-14.8 $x10^3/\mu L$, absolute lymphocyte count 2.34-7.11 $x10^3/\mu L$, platelet count 192-646 $x10^3/\mu L$, or haemoglobin 8.5-12g/dL);
- Administration of immunoglobulins and/or any blood products within the three months preceding the first study vaccination or planned administration during the study period;
- Simultaneous participation in any other interventional clinical trial;
- Acute or chronic pulmonary, cardiovascular, hepatic, renal or neurological condition, malnutrition, or any other clinical findings that in the opinion of the clinical investigator, may increase the risk of participating in the study;
- Other condition that in the opinion of the clinical investigator would jeopardize the safety or rights of a participant in the trial or would render the participant unable to comply with the protocol.

3.5 Treatment Assignment Procedures

3.5.1 Randomization Procedures

Individual participants will be randomized to receive either 30µg or 100µg of GMZ2 or rabies vaccine. Randomization to any of the three groups will be done based on a list of eligible participants identified after screening is completed. The randomization list generated by the statistician will ensure that the random allocation has a fairly even distribution of sex and age in the groups. The randomization list will contain sequential codes linking a study number to a vaccine assignment. Study numbers will be assigned to participants of each cohort in the order in which they are enrolled in the trial. Access to the randomization list will be exclusively limited to the vaccine administrator/pharmacist(s) and the study statistician. These individuals will be unblinded and will not be involved in any of the study participants' evaluation. The independent Local Safety Monitor will also keep one set of the randomization code in a sealed envelope, for the event when emergency unblinding may be required.

3.5.2 Masking Procedures

Measures will be taken to keep participants and clinical investigators (including the PI) and all other staff involved in measuring study outcomes blinded to treatment allocation. The GMZ2 vaccine and the rabies vaccine will have different appearances. Therefore, blinding of the individual preparing the study vaccine will not be possible. Since the candidate and comparison vaccines can be distinguished by appearance, the vaccine preparation area and the vaccination area will be physically separated. The vaccine administrator and assistant, both experienced and trained pharmacists, will be exclusively dedicated to vaccine preparation.

The syringe barrels will be covered with opaque tape. Vaccinators will be physicians and nurses not directly involved in evaluation/follow up of participants, so that even if they realize which vaccine they are injecting, they will not be involved in the assessment of adverse events following vaccination. Each participant will be vaccinated in a closed room out of view of anyone other than the vaccinators, so that each participant sees only the syringe he is injected with and do not see other participants being injected. Evaluation of immediate adverse events will be done in the observation room by clinicians who will remain blinded to the vaccine administered.

3.5.3 Reasons for Withdrawal

The following criteria will be checked at each visit. If any become applicable during the study, the participant will not be required to discontinue the study, but a separate immunogenicity analysis may be done that excludes these individuals.

- Use of any investigational drug or vaccine other than the study vaccine(s) during the study period.

- Chronic administration (defined as more than 14 days) of any dose level of immuno-suppressants or other immune-modifying drugs during the study period and chronic daily use of inhaled steroids. Intermittent use of inhaled and topical steroids is allowed.

- Administration of a vaccine not foreseen by the study protocol during the period starting from 30 days before the first study vaccination and ending 30 days after the third vaccination.

- Administration of immunoglobulins and/or any blood products up to 30 days after the last study vaccination.

The following criteria will be checked prior to each vaccination and are contraindications to further vaccination. However, the study participants will be encouraged to continue to participate in the surveillance schedule for safety evaluation.

- Systemic hypersensitivity reaction following administration of the study vaccine. Severe (i.e., Grade 3) local reactions will be evaluated to determine whether or not further study vaccinations should be administered.

Participants may also withdraw voluntarily from receiving the study intervention upon request for any reason.

3.5.4 Handling of Withdrawals

Every effort will be made to collect safety data on any participant discontinued because of an AE or SAE by continuing the safety follow-up procedures. If voluntary withdrawal occurs, the participant will be asked to continue scheduled evaluations and be given appropriate care under medical supervision until the symptoms of any AE resolve or the participant's condition becomes stable. If possible, participants who leave the study area will be traced and visited by clinical investigators to collect all possible safety follow-up data.

3.5.5 Termination of Study

The trial may be terminated by the sponsors or the PI due to development of serious laboratory toxicities or other major safety concern identified during interim safety analyses by the DSMB or at any other time.

"Any subject who does not finish the trial must be classified based on the following categories: discontinuation, voluntary withdrawal (or drop-out), lost to follow-up".

3.5.6 Definitions

Discontinuation by the investigator: the decision to terminate the participant's participation was taken by the investigator e.g. for medical reasons (for participant's safety), for practical reasons (non respect of study schedule), serious adverse event, etc

Voluntary withdrawal or Drop-out: a participant included in the trial is said to have dropped out after legal consenting guardian deciding, on his/her own volition, to terminate participation of the child in the trial.

The participant's guardian may decide to withdraw from the trial at any time. The investigator should make sure, however, that withdrawal was not due to an adverse event. The reason for withdrawal should be noted in the space provided for this purpose in the Case Report Form (CRF).

Lost to follow-up: when the participant could not be found in spite of the investigator's efforts to locate him. If the participant is rediscovered later on, efforts would be made to collect all possible safety information.

4 INTERVENTION/INVESTIGATIONAL PRODUCT

4.1 Study Product Description

4.1.1 Description

The GMZ2 is a recombinant vaccine. The fusion protein of vaccine interest is a hybrid of MSP3 and GLURP antigens. After expression of the protein in *Lactococcus lactis system*, GMZ2 is purified and lyophilised. The vaccine is presented in multi-dose vials, and has an aspect of white amorphous powder.

4.1.2 Formulation

Active ingredient: (GMZ2): 129, 50 µg/ml (1 ml/vial)
Other ingredients: 10 mM KH_2PO_4 + 137 mM NaCl + 0,6% sucrose, pH 6,5

4.1.3 Manufacturer

The GMZ 2 vaccine has been manufactured by Henogen S.A in Belgium.

4.1.4 Preparation

The vaccine will be reconstituted with adjuvant. Appropriate preparation protocol will be developed that will constitute SOP for preparing 0.5mL solutions that contain either 30µg or 100µg depending on vaccine allocation as dictated by study ID numbers. The reconstitution with adjuvant will be performed under sterile conditions. The adjuvanted solution will be aliquoted in disposable vanish point syringe for a single use with a 25 mm, 23 gauge single-use needle.

4.1.5 Aluminium hydroxide formulation:

Alhydrogel® is a crystalline aluminium oxyhydroxide AlO_2H, known mineralogically as boehmite. The structure consists of corrugated sheets of aluminium octahedra. The concentration used for the candidate vaccine is 1.7mg/ml Al^{3+}.

4.1.6 Precaution for use

A separate sterile vanish point syringe will be used for each individual trial participant to prevent transmission of infectious agents. Before injection, the site of injection will be cleansed with a suitable antiseptic and the entire content of each syringe should be used. Do not inject in a blood vessel. As for any injectable vaccine, appropriate equipment will be available in case of immediate allergic reactions.

4.2 Vaccination

Enrolled participants will receive three injections of a vaccine by intramuscular route into the deltoid muscle region, left arm at the first and third injection, and right arm will be given the second injection. Caution will be taken to ensure that the injection does not go into a blood vessel. The site of injection will be recorded on the case report form.

4.3 Prior hand Concomitant Therapy

Immunosuppressive or immuno-modulator treatments are exclusionary criteria. If it happens to be necessary to use one of them during the trial period, the data of the concerned volunteers will be considered as deviation to the protocol. Antipyretics or analgesics will not be allowed as preventive treatment of pain or fever, before the vaccination.
Other vaccinations will not be allowed during a three month period before the first trial vaccine injection and within six months after the third trial vaccine injection. If a vaccination is necessary during the banning time window, the data of the subjects will be considered as deviation to the protocol.
For all other treatment, the following data will be specified and recorded on Case Report Forms:

- Trade name and generic name
- Total daily dose
- Start and stop dates (and, if appropriate, timing requirements within the day: AM, PM, post-shot)
- Indication

4.4 Management of Vaccines- GMZ2

Labelling and Packaging

The multi-dose vaccine vials will be labelled with a standard label, mentioning that the vaccine use is restricted to clinical trial.

The reconstituted vaccines in syringes will be labelled using the model below:

___µg dose (Group __)<br><br>Trial: GMZ2_03_08<br>(__) doses Intramuscular (1 dose = 0.5 ml)<br>Store between +2 and +8° **C.**<br>Expiry (date & time): (___hours from mixing time)<br>Contact person: Dr. Saadou Issifou<br>Tel : +241 07847740<br>For clinical trial use only

4.5 Storage and Shipment Conditions

A qualified pharmacist will be personally responsible for product management. The vaccines will be shipped to the study centre according to the pre-determined schedule which will be based on estimated study start date, and only after the written ethics approval for the study has been received at AMANET.
The pharmacist will be expected to return to the Sponsor a completed dispatch note which will be attached to the package, as acknowledgement of receipt. The person in charge of product receipt will check that the cold chain was maintained during shipment. In case of a problem, he/she should alert the Clinical Monitor immediately. The acknowledgement of receipt will be

dated and signed by the person in charge of product management. One copy will be kept archived, the other copy will be returned to the trial sponsor AMANET.

GMZ2 vaccine will be stored at a temperature of -20°C. Reconstituted vaccines will be stored at temperatures ranging from +2°C to +8°C (in a refrigerator). Temperature will be monitored daily and documented on an appropriate form during the entire trial. The reconstituted vaccines should be administered within 24hrs.
In case of deep freezing or accidental disruption of the cold chain, vaccines should never be administered and the investigator or the responsible person should contact the sponsor immediately to receive further instructions.
The rabies vaccine (Verorab) will be stored according to manufacturer’s instructions between +2 and +8°C.

4.6 Accountability

Products must be kept in a secure place. The pharmacist in charge of product management will maintain records of the product's delivery to the trial site, the inventory at the site, the dose(s) given to each subject, and the return of unused doses to the sponsor. The applicable sponsor vaccine accountability log shall be kept up to date at all times.
Should the investigator run out of product doses during the trial, he/she should alert the Clinical Monitor who will undertake the necessary steps to ensure that extra doses available in time for the next vaccinations.

4.7 Return of Unused Products

Unused and/or open products will be returned to AMANET at the end of the vaccination period together with the form "Return of unused and/or open products" in accordance with the Monitor's instructions.

4.8 Concomitant Medications/Treatments

At each study visit/contact, the investigator will question the guardians about any medication that may have been given to the participant, including traditional medicines. Concomitant medication, including any vaccine other than the study vaccines, and any other medication relevant to the protocol, including any specifically contraindicated or administered during the period starting from one week before each study vaccination and ending one month (maximum 30 days) after will be recorded in the CRF with trade name and/or generic name of the medication, medical indication, start and end dates of treatment.

5 PARTICIPANT PROCEDURES

5.1 Screening Day –14 to –1

The accepted process of reaching the community that has been established over the years between MRU and Lambaréné community will be followed. This requires that after the protocol is approved by the authorities, special information dissemination meetings are held starting with the key civic and traditional leaders. Awareness campaigns are then undertaken including peer communication groups before the reaching the stage of small groups of potential participants, and finally individuals.

After community information is disseminated as described, all interested guardians of potential participants will be invited to visit the study clinic on a specific date. They will receive oral and written explanation of the study, after which the screening consent will be obtained from those willing to participate. After the study has been explained to the parents/guardians of potential participants, they will be free to leave and return later with their decision; this will allow time for them to discuss the study with their family and carefully consider their involvement in the study.

All screening tests, medical history and examinations will be performed only after screening consent is obtained. Clinical Investigators will generally handle acute, simple conditions such as malaria or other acute infections. More complicated or chronic conditions, such as chronic renal or heart disease, will be referred to appropriate sources of medical care.
It is estimated that 50-60 children will be screened in order to obtain a list of at least 40 eligible participants. Participants will be recruited among children residing in Lambaréné.

A screening form will be prepared for each participant and will later become part of the CRF for participants enrolled in the vaccine trial. A unique screening identification number will be assigned to each study participant. A medical history will be taken with special attention to recurrent infections to suggest immune suppression, previous history of splenectomy and prior vaccine reactions. Physical examination and laboratory screening tests will include: full blood count (FBC), creatinine, total bilirubin, ALT, AST, alkaline phosphatase. A participant who meets any of the exclusion criteria will be excluded. Participants excluded from this study because of significant abnormalities will be managed initially by Clinical Investigators and may be referred to the hospital for further evaluation as may be necessary. Screening tests will be completed within 14 days prior to entry into the study. Laboratory studies may be conducted at other times during the course of the trial if the investigators judge it necessary for the safety of the participant.

Visit 1 (may take place over more than one visit)

- Written informed consent for screening
- Medical history of participant
- Complete physical examination
- Collect 5 ml venous blood sample to measure:

- Haematology: Full Blood Count (FBC) which includes: red blood cells, platelets, white blood cell count, lymphocyte count, haemoglobin, mean corpuscular volume and mean corpuscular haemoglobin concentration, haematocrit (2.5 ml).
- Biochemistry: serum creatinine, total bilirubin, ALT, AST, alkaline phosphatase (2.5 ml).
- Blood smear for malaria parasites
- Check for inclusion and exclusion criteria
- Written informed study consent for vaccination

5.2 Enrolment/Baseline

When a total of 40 eligible participants are screened, a list with their screening numbers, dates of birth and sex will immediately be generated and transmitted to the study statistician based at the London School of Hygiene and Tropical Medicine. The statistician will then generate a randomisation code list that allocates study ID numbers linked to vaccine allocation. This list will be generated to ensure that an even distribution of age and sex is achieved in each of the three study groups. The statistician will then send the randomisation code list directly to the study pharmacist and local safety monitor via email with a secure password to access the list.

At the site study numbers will be assigned to participants of each cohort in the order in which they come to MRU on the day of the first vaccination. Immediately after the first vaccination, each study participant will receive a photo ID card and a copy of the photo ID will be attached to the CRF folder for each participant. The vaccine and dose that are assigned during the first vaccination will be maintained for second and third vaccinations.

Access to the randomization list will be exclusively limited to the study drug manager(s)/pharmacist(s) and study statistician. These individuals are unblinded and will not be involved in study participants' further evaluation. The Local Safety Monitor will also keep one set of the randomization code in a sealed envelope, in the event that emergency unblinding is required. The reason for any unblinding will be documented as well as the steps to be taken.

5.3 Vaccination process

Before each vaccination, exclusion criteria will be reviewed and verified. A history-directed physical examination will be done and temperature and baseline general symptoms/signs will be recorded.
After the participant's identity is checked by comparing his name with the list of eligible volunteers, vaccination will be administered by intramuscular injection into the left deltoid muscle. If any local impairment prevents administration of the vaccine into the preferred deltoid for that particular vaccination, the vaccine may be administered into the opposite deltoid, and accordingly documented. Vaccination will be done on study days 0, 28 +/- 3 and 56+/- 3.

Day 0: Vaccination 1 - *Visit 2*

Before vaccination the following steps will be taken**:**

- Countercheck to confirm the identity of the participant
- Review screening laboratory test results
- Review inclusion/exclusion criteria and check of contraindications/precautions
- Record any complaints, symptom-directed physical examination, and examination of the injection site(s) for any abnormalities
- Record vital signs: tympanic temperature

- Collect 7.5 ml venous blood sample to measure:
  - FBC (EDTA) 2.5 ml.
  - Biochemistry, WB, ELISA, and GIM (Serum) 2.5 ml.
  - Peripheral blood mononuclear cells (PBMC) for memory B-cell ELISPOT 2.5 ml
- Give time to calm the child after the blood draw
- Administer the vaccination dose 1 according to SOP
- Assignment of unique study number

After vaccination the following steps must be taken:

- Observe for a minimum of 30 minutes
- Record tympanic temperature
- Assessment of the immediate local and systemic solicited events
- Prepare an ID card containing participant's unique study number and photo
- Ensure that the copy of the ID photo is attached to the participant's CRF
- Provide further information on when the next visit should be
- Instruct the guardian to return to the MRU centre immediately should the participant manifest any signs or symptoms they perceive as important.

Days 1: Post-vaccination 1 surveillance clinic visit
Visit 3

- Verify and confirm ID of the participant
- Review history for any adverse events
- Targeted physical examination for solicited local and systemic symptoms
- Record tympanic temperature
- Record other signs/symptoms
- Give details of next visit and remind guardian on what to do if case of any problem

Day 3: Post-vaccination 1 surveillance clinic visit
Visit 4

- Verify and confirm ID of the participant
- Review history for any adverse events
- Targeted physical examination for solicited local and systemic symptoms
- Record tympanic temperature
- Record other signs/symptoms
- Give details of next visit and remind guardian on what to do if case of any problem

Days 2- 6: Post-vaccination 1 surveillance home visits on days 2, 4, 5 and 6
Field worker home visits

- Complete the field worker home visit card information
- Solicited symptoms
- Record tympanic temperature
- Record any adverse events occurring after the previous study vaccination
- Provide details on next visit and what to do in case of any problem

Day 7± 1 day: Post-vaccination 1 surveillance visit
Visit 5

- Verify and confirm ID of the participant
- Review history for any adverse events
- Targeted physical examination for solicited local and systemic symptoms
- Record tympanic temperature
- Record other signs/symptoms
- Give details of next visit and remind guardian on what to do if case of any problem

Day 14± 2 days: Post-vaccination 1 surveillance visit
Visit 6

- Verify and confirm ID of the participant
- Review history for any adverse events
- Targeted physical examination for solicited local and systemic symptoms
- Record tympanic temperature
- Record other signs/symptoms
- Give details of next visit and remind guardian on what to do if case of any problem

Day 28± 3 days: 4 weeks post-vaccination 1 surveillance visit and vaccination 2
Visit 7

Before vaccination the following steps will be taken**:**
- Countercheck to confirm the identity of the participant
- Review inclusion/exclusion criteria and check of contraindications/precautions
- Record any complaints, symptom-directed physical examination, and examination of the injection site(s) for any abnormalities
- Record vital signs: tympanic temperature
- Collect 5 ml venous blood sample to measure:
    - FBC (EDTA) 2.5 ml.
    - Biochemistry, WB, ELISA, and GIM (Serum) 2.5 ml.
- Give time to calm the child after the blood draw
- Administer the vaccination dose 2 according to SOP

After vaccination the following steps must be taken:
- Observe for a minimum of 30 minutes
- Record tympanic temperature
- Assessment of the immediate local and systemic solicited events
- Provide further information on when the next visit should be
- Instruct the guardian to return to the MRU centre immediately should the participant manifest any signs or symptoms they perceive as important.

Days 29: 1 day, post-vaccination 2 surveillance clinic visit
Visits 8

- Verify and confirm ID of the participant

- Review history for any adverse events
- Targeted physical examination solicited local and systemic symptoms
- Record tympanic temperature
- Record other signs/symptoms
- Give details of next visit and remind guardian on what to do if case of any problem

Days 31: 3 days post-vaccination 2 surveillance clinic visit
Visit 9

- Verify and confirm ID of the participant
- Review history for any adverse events
- Targeted physical examination for solicited local and systemic symptoms
- Record tympanic temperature
- Record other signs/symptoms
- Give details of next visit and remind guardian on what to do if case of any problem

Days 30-34 Post-vaccination surveillance home visits on days 30, 32, 33, and 34
Field worker home visits

- Complete the field worker home visit card information
- Solicited symptoms
- Record tympanic temperature
- Record any adverse events occurring after the previous study vaccination
- Provide details on next visit and what to do in case of any problem

Day 35± 1 day: 7 days post-vaccination 2 surveillance visit
Visit 10

- Verify and confirm ID of the participant
- Review history for any adverse events
- Targeted physical examination for solicited local and systemic symptoms
- Record tympanic temperature
- Record other signs/symptoms
- Give details of next visit and remind guardian on what to do if case of any problem

Day 42± 2 days: 14 days post-vaccination 2 surveillance visit
Visit 11

- Verify and confirm ID of the participant
- Review history for any adverse events
- Targeted physical examination for solicited local and systemic symptoms
- Record tympanic temperature
- Record other signs/symptoms
- Give details of next visit and remind guardian on what to do if case of any problem

Day 56± 3 days: 4 weeks post-vaccination 2, surveillance visit and vaccination 3
Visit 12

Before vaccination the following steps will be taken**:**

- Countercheck to confirm the identity of the participant
- Review inclusion/exclusion criteria and check of contraindications/precautions
- Record any complaints, symptom-directed physical examination, and examination of the injection site(s) for any abnormalities
- Record vital signs: tympanic temperature
- Collect 5 ml venous blood sample to measure:
    - FBC (EDTA) 2.5 ml.
    - Biochemistry, WB, ELISA, and GIM (Serum) 2.5 ml.
- Give time to calm the child after the blood draw
- Administer the vaccination dose 3 according to SOP

After vaccination the following steps must be taken:

- Observe for a minimum of 30 minutes
- Record tympanic temperature
- Assessment of the immediate local and systemic solicited events
- Provide further information on when the next visit should be
- Instruct the guardian to return to the MRU centre immediately should the participant manifest any signs or symptoms they perceive as important.

Days 57: 1 day post-vaccination 3 surveillance visits
Visit 13

- Verify and confirm ID of the participant
- Review history for any adverse events
- Targeted physical examination for solicited local and systemic symptoms
- Record tympanic temperature
- Record other signs/symptoms
- Give details of next visit and remind guardian on what to do if case of any problem

Days 59: 3 days post-vaccination 2 surveillance clinic visit
Visit 14

- Verify and confirm ID of the participant
- Review history for any adverse events
- Targeted physical examination for solicited local and systemic symptoms
- Record tympanic temperature
- Record other signs/symptoms
- Give details of next visit and remind guardian on what to do if case of any problem

Days 58- 62 Post-vaccination surveillance home visits on days 58, 60, 61, and 62
Field worker home visits

- Complete the field worker home visit card information
- Solicited symptoms

- Record tympanic temperature
- Record any adverse events occurring after the last study vaccination
- Provide details on next visit and what to do in case of any problem

Day 63± 1 days: 7 days post-vaccination 3 surveillance visit
Visit 15

- Verify and confirm ID of the participant
- Review history for any adverse events
- Targeted physical examination for solicited local and systemic symptoms
- Record tympanic temperature
- Record other signs/symptoms
- Give details of next visit and remind guardian on what to do if case of any problem

Day 70± 2 days: 14 days post-vaccination 3 surveillance visit
Visit 16

- Verify and confirm ID of the participant
- Review history for any adverse events
- Targeted physical examination for solicited local and systemic symptoms
- Record tympanic temperature
- Record other signs/symptoms
- Give details of next visit and remind guardian on what to do if case of any problem

Day 84 ± 3 days: 4 weeks post-vaccination 3, surveillance visit
Visit 17

- Countercheck to confirm the identity of the participant
- Record any complaints, symptom-directed physical examination, and examination of the injection site(s) for any abnormalities
- Record vital signs: tympanic temperature
- Assessment of the protocol local and systemic solicited events
- Collect 7.5 ml venous blood sample to measure:
    - FBC (EDTA) 2.5 ml.
    - Biochemistry, WB, ELISA, and GIM (Serum) 2.5 ml.
    - Peripheral blood mononuclear cells (PBMC) for memory B-cell ELISPOT 2.5 ml

- Provide further information on when the next visit should be
- Instruct the guardian to return to the MRU centre immediately should the participant manifest any signs or symptoms they perceive as important.

Follow up visits: Post-vaccination surveillance home visits on days 140, 224, and 308
Field worker home visits

These visits will be undertaken to maintain contact with the participants during the long term follow up period, to collect any adverse event information that may have been missed, and remind them of the next protocol activity.

- Complete the field worker home visit card information
- Record any adverse events occurring since the last contact
- If any event has occurred that may be deemed to be serious, bring back the participant to the clinic for investigator evaluation
- Provide details on next visit and what to do in case of any problem.

Day 365± 14 days: Study completion visit
Visit 18

This visit constitutes the last protocol foreseen visit for each participant. The following steps will be taken during this visit.

- Countercheck to confirm the identity of the participant
- Record any complaints, symptom-directed physical examination, and examination of the injection site(s) for any abnormalities
- Record vital signs: tympanic temperature
- Collect 7.5 ml venous blood sample to measure:
  - FBC (EDTA) 2.5 ml.
  - Biochemistry, WB, ELISA, and GIM (Serum) 2.5 ml.
  - Peripheral blood mononuclear cells (PBMC) for memory B-cell ELISPOT 2.5 ml
- Give time to calm the child after the blood draw
- Review the patient records for any missing information
- If there is any adverse event, it must be treated according to the existing standard of care, and follow up till resolution.
- Give estimated time for when results of the study will be communicated back to the community
- Reassure them maintaining confidentiality, especially of their identity.

5.4 Follow-up

After each vaccination, participants will be observed for local and systemic reactions for a minimum of 30 minutes. Signs and symptoms will be solicited and recorded by the investigators. Participants will further be followed up by trained field workers during the next six days following each vaccination. During these field worker visits, information on the medical condition of the participants will be obtained using a standardised field worker card. Any adverse events occurring during the follow up period will be notified to the investigators and followed until resolution or stabilisation. If any symptom persists beyond the 14-day surveillance period the participant will be followed daily until resolution of the adverse event.
Every effort will be made to ensure compliance with visits. If a participant does not appear for a scheduled clinic visit, the local guide will visit him again and accompany the participant to the clinic centre. If a serious adverse event (SAE) has occurred, appropriate reporting procedures must be followed as described in section 6.
The following is the summary schedule for the planned field worker visits:

- Post vaccination 1: Days 2, 4, 5 and 6

- Post vaccination 2: Days 30, 32, 33 and 34
- Post vaccination 3: Days 58, 60, 61 and 62
- Long-term follow up: Days 140, 224 and 308

5.5 Unscheduled Visits

Participants will be advised to report to the MRU anytime they feel unwell, regardless of their perceived cause. At such unscheduled visits, a history and physical examination, clinical laboratory tests including malaria smear if indicated, documentation of any AEs, and any other medically indicated diagnostic or therapeutic procedures will be done. These will be recorded as observations in the participant's study record and if the physician finds them to be adverse events, the appropriate CRF sections will be completed.

6 ADVERSE EVENTS AND REPORTING PROCEDURES

6.1 Definitions

Definitions for the terms adverse event (or experience), adverse reaction, and unexpected adverse reaction have previously been agreed to through the ICH-GCP guidelines and by consensus of the more than 30 Collaborating Centres of the WHO International Drug Monitoring Centre (Uppsala, Sweden). Although those definitions can pertain to situations involving clinical investigations, some minor modifications are necessary, especially to accommodate the pre-approval, development environment.
The following definitions, with input from the WHO Collaborative Centre, have been agreed:

6.1.1 Adverse Event (or Adverse Experience AE)

An AE is any untoward medical occurrence in a clinical investigation subject, temporally associated with the use of a medicinal product, whether or not considered related to the medicinal product.
An AE can therefore be any unfavourable and unintended sign (including an abnormal laboratory finding), symptom, or disease (new or exacerbated) temporally associated with the use of a medicinal product. For marketed medicinal products, this also includes failure to produce expected benefits (i.e. lack of efficacy), abuse or misuse.

6.1.2 Adverse Drug Reaction (ADR)

In the *pre-approval clinical* experience with a new medicinal product or its new usages, particularly as the dose(s) may not be established, all noxious and unintended responses to a medicinal product related to any dose should be considered adverse drug reactions.
The phrase "responses to a medicinal product" means that a causal relationship between a medicinal product and an adverse event is at least a reasonable possibility, i.e., the relationship cannot be ruled out.

6.1.3 Unexpected Adverse Drug Reaction

An adverse drug reaction, the nature or severity of which is not consistent with the applicable product information (e.g. Investigator's Brochure for an unapproved investigational medicinal product) is considered as an unexpected adverse drug reaction.
When there is a Serious Adverse Event (SAE) that is unexpected and associated with the use of the medication (Suspected Unexpected Serious Adverse Reaction (SUSAR)), and which occurs during or after treatment, an “expedited report” is to be submitted to the regulatory authority.
Medical and scientific judgment should be exercised in deciding whether “expedited reporting” is appropriate in other situations, such as important medical events that may not be immediately life-threatening or result in death or hospitalisation but may jeopardise the patient or may require intervention to prevent one of the other outcomes listed in the definition above. These should also usually be considered serious.
The purpose of “expedited reporting” is to make regulators, Investigators, and other appropriate people aware of new, important information on serious reactions. Therefore, such reporting will generally involve events previously unobserved or undocumented, and a guideline is needed on how to define an event as "unexpected" or "expected" (expected/unexpected from the

perspective of previously observed, not on the basis of what might be anticipated from the pharmacological properties of a medicinal product).

6.1.4 Serious Adverse Event

During clinical investigations, adverse events may occur which, if suspected to be medicinal product-related (adverse drug reactions), might be significant enough to lead to important changes in the way the medicinal product is developed (e.g., change in dose, population, needed monitoring, consent forms). This is particularly true for reactions which, in their most severe forms, threaten life or function. Such reactions should be reported promptly to regulators. Therefore, special medical or administrative criteria are needed to define reactions that, either due to their nature ("serious") or due to the significant, unexpected information they provide, justify expedited reporting. To ensure no confusion or misunderstanding of the difference between the terms "serious" and "severe," which are not synonymous, the following note of clarification is provided:
The term "severe" is often used to describe the intensity (severity) of a specific event (as in mild, moderate, or severe myocardial infarction); the event itself, however, may be of relatively minor medical significance (such as severe headache). This is not the same as "serious," which is based on patient/event outcome or action criteria usually associated with events that pose a threat to a patient's life or functioning. Seriousness (not severity) serves as a guide for defining regulatory reporting obligations
A serious adverse event (experience) or reaction is any untoward medical occurrence that at any dose:

- results in death,
- is life threatening,
- requires inpatient hospitalisation or prolongation of existing hospitalisation,
- results in persistent or significant disability/incapacity, or
- is a congenital anomaly/birth defect.

NOTE: *The term "life-threatening" in the definition of "serious" refers to an event in which the patient was at risk of death at the time of the event; it does not refer to an event which hypothetically might have caused death if it were more severe*.

Medical and scientific judgment should be exercised in deciding whether expedited reporting is appropriate in other situations, such as important medical events that may not be immediately life-threatening or result in death or hospitalisation but may jeopardise the patient or may require intervention to prevent one of the other outcomes listed in the definition above. *These should also usually be considered serious.*

6.1.5 Clinical laboratory parameters and other abnormal assessments qualifying as adverse events and serious adverse events

Abnormal laboratory findings (e.g., clinical chemistry, haematology, and urinalysis) or other abnormal assessments that are judged by the investigator to be **clinically significant** will be recorded as AEs or SAEs if they meet the definition of an AE, as defined in Section 6.1.1 or SAE, as defined in Section 6.1.4 Clinically significant abnormal laboratory findings or other abnormal assessments that are detected during the study or are present at baseline and significantly worsen following the start of the study will be reported as AEs or SAEs.
The investigator will exercise his or her medical and scientific judgement in deciding whether an abnormal laboratory finding or other abnormal assessment is clinically significant.

6.2 Safety Monitoring Plan

6.2.1 Role of the Safety Monitor:

The Local Safety Monitor (LSM) will be an experienced clinician qualified to evaluate safety data from clinical trials of vaccines. He/she will not be directly involved in the clinical trial and will be based in Lambaréné. All SAEs will be reported to him/her. In exceptional circumstances, for example a death possibly related to vaccination, or under conditions as outlined in below in the safety monitoring plan, he/she will have the authority to suspend vaccination pending discussion with the sponsor and collaborators. He will hold the code-break information and under conditions as outlined below, he will have the authority to unblind individual subjects. All unblinding should be reported immediately to AMANET. The LSM will also pass on his reports to the Data Safety Monitoring Board (DSMB) regularly, during the process of the trial.
Thus the LSM's role will include:

- Acting as the study participants' advocate
- Promptly communicating relevant safety information to the DSMB
- Providing advice to the investigators on whether a set of clinical circumstances in a study warrants formal notification to AMANET and DSMB
- Providing clinical advice on any illness in study participants especially in circumstances in which treatment might influence the course of the trial
- Review all SAEs as outlined above

6.2.2 The Data Safety Monitoring Board (DSMB)

An independent DSMB will be constituted by AMANET to review safety data at specified intervals. The membership of the DSMB will include expertise in clinical vaccine trials in malaria endemic settings, paediatrics, and biostatistics. The role of the DSMB is to provide safety oversight over the conduct of the trial. It will review safety continuously during the trial and will advise on progression of the trial. The DSMB will hold conference calls to review the safety data generated from the trial as generated by the investigators using a standard report template up to specific points. The purpose of these conference calls will be to review the accumulated safety data in order to advise whether or not the study integrity remains intact and whether or not there are any safety concerns worth early notice. In the event that the DSMB identifies a safety concern, the collaborative group including the investigators and AMANET and EMVI will review all safety data in order to decide the next step. Senior investigators and representatives from AMANET will be invited to but not obligated to participate in the conference calls during open sessions, which will be followed by closed sessions including only DSMB members.

The investigator will inform the DSMB of:

- All subsequent protocol amendments, informed screening or study consent form changes or revisions of other documents originally submitted for review
- Serious adverse events (SAEs) and Grade 3 adverse experiences occurring during the study, regardless of relationship to the study vaccine
- New information that may affect adversely the safety of the participants or the conduct of the study.

The DSMB may recommend that AMANET put the study on temporary hold pending review of potential safety issues. The DSMB will request additional information from the Principal Investigator as needed and will request any appropriate statistical calculations to support discussions with AMANET. All documentation provided to members of the DSMB for information and review will be treated in a confidential manner. The detailed terms of reference and modus

operendi for the DSMB is contained in the separate DSMB charter, which constitutes part of the documentation for this study.

6.2.3 Holding Rules

Vaccination will be performed in a randomised plan. After each vaccination, safety data will be provided in tables by the data manager. The safety tables will be reviewed by the DSMB in a blinded manner.
If there are no safety issues noted, the study will proceed as planned. However, if based on the review of the safety data tables provided, 50% of participants in a particular are found to have developed a Grade 3 adverse event, related to vaccination and persisting at Grade 3 for > 48 hours during the 14 follow-up days after vaccination, a decision of individual un-blinding may then be made by DSMB through the Local Safety Monitor. Subsequent vaccination of that group will be put on hold pending discussion with the investigator, and the sponsor.
Within five working days of the Local Safety Monitor placing vaccination of a group on hold, the Sponsor will organise a meeting (via teleconference, or face-to-face) to review and discuss the safety data and the events leading to the hold order. At least two working days prior to this meeting, the Sponsor will disseminate copies of all relevant safety data to all meeting participants.

Activation of the Holding Rules requires a thorough review by the DSMB of blinded reactogenicity and safety data and discussion with the investigator, and the sponsor.

6.2.4 Process for restarting vaccination

The vaccination of a group may be put on hold. Continued vaccination of that group may restart only if AMANET expressively gives authorisation to the Principal Investigator to a resumption of vaccination.

6.2.5 Process for stopping vaccination of a group or of the trial

In the event that vaccination of a particular group is stopped, the Sponsor will inform the IEC/IRB through the investigator. A report will be written detailing the rationale used for reaching this decision.

6.3 Safety Data Collection and Management Procedures

6.3.1 Expected Adverse Vaccine Reactions

As for any adjuvanted vaccine, local reactions are expected. Systemic reactions are less often observed; however, a standardized data collection of adverse reactions will be organized.
So far, clinical experience with aluminium hydroxide has shown that the most frequent local reactions are pain, induration, erythema, swelling at the site of injection. Those reactions are usually mild and transient. There is a lack of information concerning the expected systemic reactions; therefore, a special attention will be given to general signs, i.e. fever, irritability or fussiness, drowsiness, and loss of appetite.
In case of a severe local skin reaction, uni-lateral or contra-lateral, a skin biopsy may be needed as part of the clinical evaluation.

6.3.2 Safety Data Collection

All the Adverse Events, whether observed by the Clinical Investigator or by the parent/guardian of the participant, will be carefully and accurately documented in the CRF by the Clinical Investigator. For each event/reaction the following details will be recorded:

1. Description of the event(s)/reaction(s),
2. Date and time of occurrence,
3. Duration,
4. Intensity,
5. Relationship with the vaccine,
6. Action taken, including treatment,
7. Outcome.

Safety assessments will be obtained and recorded by the Investigator. He/She will not know which vaccine formulation the subject has received. “Solicited” and “unsolicited” reactions/events will be actively followed during 30 minutes after each injection, and during the following 14 days. The table below summarizes the solicited local and systemic reactions that will be sought after and how intensity will be graded.

Summary table for solicted local and systemic events with grading system

Adverse Event	Intensity grade	Parameter
Pain at injection site	0	Absent
	1	Minor reaction to touch
	2	Cries/protests on touch
	3	Cries when limb is moved/spontaneously painful
Swelling at injection site*		Record greatest surface diameter in mm
Induration at injection site*		Record greatest surface diameter in mm
Erythema at injection site*		Record greatest surface diameter in mm
Contra-lateral reaction*		Record greatest surface diameter in mm
Pruritus at injection site	0	Absent
	1	Easily tolerated by the subject, causing minimal discomfort and not interfering with everyday activities.
	2	Sufficiently discomforting to interfere with normal everyday activities.
	3	Prevents normal, everyday activities.
Fever	0	Tympanic temperature < 38°C
	1	$38 \leq$ and $<38.5\,^{o}C$
	2	$38.5 \leq$ and $<39\,^{o}C$
	3	$\geq 39\,^{o}C$
Irritability/Fussiness	0	Behavior as usual
	1	Crying more than usual/ no effect on normal activity
	2	Crying more than usual/ interferes with normal activity
	3	Crying that cannot be comforted/ prevents normal activity
Drowsiness	0	Behavior as usual
	1	Drowsiness easily tolerated
	2	Drowsiness that interferes with normal activity
	3	Drowsiness that prevents normal activity
Loss of appetite	0	Appetite as usual
	1	Eating less than usual/ no effect on normal activity

	2	Eating less than usual/ interferes with normal activity
	3	Not eating at all
Diarrhoea	0	None
	1	More than usual, but no dehydration
	2	With some dehydration
	3	With severe dehydration

The maximum intensity of local injection site swelling, induration and erythema will be scored as follows:

0	None
1	< 25 mm
2	25$\leq$ and <50 mm
3	$\geq$ 50 mm

Reports of all reactions will be investigated and documented in the source record. Local symptoms will be ranked as mild (grade 1), moderate (grade 2) or severe (grade 3) by the size of the reaction as summarised above.

6.3.3 Time period, frequency, and method of detecting adverse events and serious adverse events

All AEs occurring within one month following administration of each dose of vaccine must be recorded on the Adverse Event form in the subject's CRF, irrespective of severity or whether or not they are considered vaccination-related.

Additionally, in order to fulfil international reporting obligations, SAEs that are related to study participation (e.g. procedures, invasive tests, a change from existing therapy) or are related to a concurrent medication will be collected and recorded from the time the subject consents to participate in the study until she/he is discharged.

The investigator will inquire about the occurrence of AEs at every visit/contact during the study and throughout the follow-up phase as appropriate.

All AEs either observed by the investigator or one of his clinical collaborators or reported by the participant’s parent/guardians spontaneously or in response to a direct question will be evaluated by the investigator. AEs occurring within one month following administration of each dose of vaccine not previously documented in the study will be recorded in the Adverse Event form within the subject's CRF. The nature of each event, date and time (where appropriate) of onset, outcome, intensity and relationship to vaccination should be established. Details of any corrective treatment should be recorded on the appropriate page of the CRF.

As a consistent method of soliciting AEs, the subject will be asked non-leading questions such as:

"Have you felt different in any way since receiving the vaccine or since the previous visit?"

AEs already documented in the CRF, i.e. at a previous assessment, and designated as “not recovered/not resolved” or “recovering/resolving” should be reviewed at subsequent visits, as necessary. If these have resolved, the documentation in the CRF should be completed.

Note: If an AE changes in frequency or intensity during the specified reporting period, a new record of the event should be entered.

When an AE/SAE occurs, it is the responsibility of the investigator to review all documentation (e.g., hospital progress notes, laboratory, and diagnostics reports) relative to the event. The investigator will then record all relevant information regarding an AE/SAE on the CRF or SAE Report Form as applicable. It is not acceptable for the investigator to send photocopies of the subject's medical records to AMANET in lieu of the appropriate completed AE/SAE pages. However, there may be instances when copies of medical records for certain cases are requested by AMANET. In such an instance, all participant identifiers will be blinded on the copies of the medical records prior to submission to AMANET.
The investigator will attempt to establish a diagnosis of the event based on signs, symptoms, and/or other clinical information. In such cases, the diagnosis should be documented as the AE/SAE and not the individual signs/symptoms.

6.3.4 Assessment of causality

The clinical investigator is obliged to assess the relationship between investigational product and the occurrence of each AE/SAE. The investigator will use clinical judgement to determine the relationship. Alternative causes, such as natural history of the underlying diseases, concomitant therapy, other risk factors and the temporal relationship of the event to the investigational product will be considered and investigated. The investigator will also consult the Investigator Brochure and/or Product Information, for marketed products, in the determination of his/her assessment.
There may be situations when a SAE has occurred and the investigator has minimal information to include in the initial report. However, it is very important that the investigator always makes an assessment of causality for every event prior to transmission of the SAE Report Form to AMANET. The investigator may change his/her opinion of causality in light of follow-up information, amending the SAE Report Form accordingly. The causality assessment is one of the criteria used when determining regulatory reporting requirements.
In case of concomitant administration of multiple vaccines, it may not be possible to determine the causal relationship of general AEs to the individual vaccines administered. The investigator should, therefore, assess whether the AE could be causally related to vaccination rather than to the individual vaccines.

All solicited local (injection site) reactions will be considered causally related to vaccination. Causality of all other AEs should be assessed by the investigator using the following question:
"*Is there a reasonable possibility that the AE may have been caused by the investigational product?*
NO: The AE is not causally related to administration of the study vaccine(s). There are other, more likely causes, and administration of the study vaccine(s) is not suspected to have contributed to the AE.
YES: There is a possible or probable cause that the vaccine(s) contributed to the AE.
Non-serious and serious AE's will be evaluated as two distinct events. If an event meets the criteria to be determined "serious" (see definition of serious adverse event), it will be examined by the investigator to the extent to be able to determine ALL contributing factors applicable to each serious adverse event.
Other possible contributors include:

- Medical history
- Other medication
- Protocol required procedure
- Other procedure not required by the protocol
- Lack of efficacy of the vaccine(s), if applicable

- Erroneous administration
- Other cause (specify)

6.3.5 Medically attended visits

For each solicited and unsolicited symptom the subject experiences, the subject will be asked if they received medical attention defined as hospitalisation, an emergency room visit or a visit to or from medical personnel (medical doctor) for any reason and this information will be recorded in the CRF.

6.3.6 Follow-up of adverse events and serious adverse events and assessment of outcome

After the initial AE/SAE report, the investigator is required to proactively follow each subject and provide further information to AMANET on the subject's condition. All AEs and SAEs documented at a previous visit/contact and designated as not recovered/not resolved or recovering/resolving will be specifically reviewed at subsequent visits/contacts.
Investigators will follow-up participants with SAEs or participants withdrawn from the study as a result of an AE, until the event has resolved, subsided, stabilized, disappeared, the event is otherwise explained, or the subject is lost to follow-up; or, in the case of other non-serious AEs, until they complete the study or they are lost to follow-up.

Clinically significant laboratory abnormalities will be followed up until they have returned to normal, or a satisfactory explanation has been provided. Additional information (including but not limited to laboratory results) relative to the subsequent course of such an abnormality noted for any subject must be made available to the Study Monitor.
AMANET may request that the investigator perform or arrange for the conduct of supplemental measurements and/or evaluations to elucidate as fully as possible the nature and/or causality of the AE or SAE. The investigator is obliged to assist. If a participant dies during participation in the study or during a recognized follow-up period, AMANET will be provided with a copy of any available post-mortem findings, including histopathology.
New or updated information will be recorded on the originally completed SAE Report Form, with all changes signed and dated by the investigator. The updated SAE report form should be resent to AMANET within 48 hours of receipt of the follow-up information.
Outcome of any non-serious AE occurring within 30 days post-vaccination (i.e. unsolicited AE) or any SAE reported during the entire study will be assessed and classified as:

1. Recovered/resolved
2. Not recovered/not resolved
3. Recovering/resolving
4. Recovered with sequelae/resolved with sequelae
5. Fatal (SAEs only)

6.3.7 Reporting of Serious Adverse Events

A. Timeframes for reporting of SAEs

SAEs will be reported promptly once the investigator determines that the event meets the protocol definition of an SAE. The investigator or designate will report to the Study Contact for Serious Adverse Event Reporting WITHIN 24 HOURS OF HIS/HER BECOMING AWARE OF THESE EVENTS. Additional or follow-up information relating to the initial SAE report is also to be reported to the Study Contact for Serious Adverse Event Reporting within 24 hours of receipt of

such information. The reporting should be principally by phone; then the investigator should immediately send the **completed SAE report** to AMANET alternatively by email or fax sending it with an acknowledgment of receipt.

B. Completion and transmission of serious adverse event reports

Once an investigator becomes aware that an SAE has occurred in a study subject, he will report the information to Study Contact for reporting SAEs within 24 hours. The SAE Report Form must always be completed as thoroughly as possible with all available details of the event, signed by the investigator (or designee). If the investigator does not have all information regarding an SAE, he/she will not wait to receive additional information before notifying Study Contact of the event and completing the form. The form will be updated when additional information is received and forwarded WITHIN 24 HOURS.

The investigator must always provide an assessment of causality at the time of the initial report as earlier described.

Initial notification via the telephone does not replace the need for the investigator to complete and sign the SAE Report Form within 24 hours.

In the event of a death determined by the investigator to be related to vaccination, sending of the fax must be accompanied by telephone call to the Study Contact for Reporting SAEs

**Study Contact for Reporting SAEs to AMANET**
Name, address: Roma Chilengi<br>Tel: +255 22 2700018<br>FAX: +255 22 2700380<br>Outside office hours<br>Mobile phone: +255 754 959367<br>Email : chilengi@amanet-trust.org

6.3.8 Regulatory reporting requirements for serious adverse events

The investigator will promptly report all SAEs to AMANET in accordance with the procedures detailed above. AMANET has a legal responsibility to promptly notify, as appropriate, both the local regulatory authority and other regulatory agencies about the safety of a product under clinical investigation. Prompt notification of SAEs by the investigator to the Study Contact for Reporting SAEs is essential so that legal obligations and ethical responsibilities towards the safety of other subjects are met.

The investigator, or responsible person according to local requirements, will comply with the applicable local regulatory requirements related to the reporting of SAEs to regulatory authorities and the IRB/IEC.

Expedited Investigator Safety Report (EISR) is prepared according to AMANET/EMVI internal policies and forwarded to investigators when necessary. An EISR is required for:

- Development compounds (i.e. compounds not marketed), if the event is serious, unexpected and has a suspected relationship to study drug treatment.
- If the expected adverse events for development compounds will be described in the Development Core Safety Information (DCSI) in the Investigator Brochure (IB).

The purpose of the EISR is to fulfil specific regulatory and ICH-GCP requirements, regarding the product under investigation.

An investigator who receives an EISR describing a SAE or other specific safety information from EMVI will file it with the Investigator Brochure and will notify the IRB/IEC, if appropriate according to local requirements.

6.3.9 Post study adverse events and serious adverse events

A post-study AE/SAE is defined as any event that occurs outside of the AE/SAE detection period defined earlier in this Section. Investigators are not obligated to actively seek AEs or SAEs in former study participants.

However, if the investigator learns of any SAE, including a death, at any time after a subject has been discharged from the study, and he/she considers the event reasonably related to the investigational product, the investigator will promptly notify the Study Contact for Reporting SAEs.

6.3.10 Treatment of adverse events

Treatment of any adverse event is at the sole discretion of the clinical investigator and according to current good medical practice. Any medication administered for the treatment of an AE should be recorded in the subject's CRF.

The clinical research facility includes private consultation rooms, a procedure room, a resuscitation suite with oxygen, suction and resuscitation kits, and a post-vaccination observation room. An ambulance with suction and oxygen will be immediately available on vaccination days, and may be accessible with a short delay on the other days. The pharmacy at the MRU will have sufficient provisions to provide participants with oral and parenteral drugs for the treatment of common illnesses (including uncomplicated and severe malaria) free of charge, using essential medicines and treatment regimens that meet or exceed standards recommended by the Gabon Ministry of Health. Blood transfusion facility is available at all times. Twenty-four hour hospitalisation and basic emergency surgery services are available at the ASH adjacent to the MRU. Twenty-four hour nursing staffing and 24-hour on call physicians will be available.

If the investigators or the Local Safety Monitor judge that a participant requires hospitalization, this will be immediately facilitated and the investigators will ensure that the best possible treatment is offered.

6.4 Lost to Follow-up Procedures

If a subject fails to appear for a follow-up examination, extensive effort (i.e., documented phone calls and certified mail) should be undertaken to locate or recall him/her or at least to determine his/her health status. These efforts should be documented in the subject's CRF and source documents.

7 EVALUATION CRITERIA

7.1 Primary Evaluation Criterion: Safety

7.1.1 Definition of the Criterion

The safety profile will be assessed on the following criteria:

- Immediate reactogenicity (reactions within 30 minutes after each injection),
- Local and systemic reactogenicity measured from Day 0 to Day 14 after each dose
- Any unsolicited adverse event resulting in a visit to a physician between each injection and one month after the third dose.
- Any Serious Adverse Event (SAE) occurring from the inclusion through out the study. The relationship of the adverse event to the study vaccine will be established by the investigator, using the following definitions: related or not related.
- Biological safety, one month after each vaccination, in reference with the baseline before the first dose, by measuring the following:
  - RBC, haemoglobin, haematocrit, platelets, total WBC;
  - serum creatinine, ASAT, ALAT, total bilirubin, alkaline phosphatase.

7.1.2 Parameters to be measured

The rate, duration and severity of signs and symptoms during the 14 days after the vaccination will be measured.

A. Local Solicited reactions

The following local reactions will be recorded and during the 14 days following the vaccination (D0 and D14 follow-up):

- **Pain:** Intensity and duration
- **Swelling:** Size (mm) and duration
- **Induration:** Size (mm) and duration
- **Erythema:** Size (mm) and duration
- **Pruritis:** presence or absence at injection site
- **Contra-lateral local reaction:** Intensity, duration and relationship with the vaccine

B. Systemic Solicited reactions

The following systemic reactions will be recorded during the 14 days following the vaccination (D0 and D14 follow-up):

- **Fever:** Intensity and duration and relationship with the vaccine
- **Irritability:** Intensity and duration and relationship to vaccine
- **Drowsiness:** Intensity, duration and relationship with the vaccine

- **Loss of appetite:** Intensity, duration and relationship with the vaccine
- **Diarrhoea :** Intensity, duration and relationship with the vaccine

C. Unsolicited adverse events

For each unsolicited event/reaction the following details will be recorded:

1. Description of the event(s)/reaction(s),
2. Date and time of occurrence,
3. Duration,
4. Intensity,
5. Relationship with the vaccine,
6. Outcome.

7.1.3 Method and Timing of Measurement

A. Local solicited reactions

The duration of the **pain** will be measured in days, and the severity scale as Grade 1 (mild): easily tolerated, does not interfere with daily life; Grade 2 (moderate): is discomfort that interferes with or limits usual daily activity; Grade 3 (severe): is disabling, inability to perform usual daily activity, including a functional limitation of the movement.

The duration of erythema, swelling, and other local reaction will also be measured in days. The size of erythema, and induration will be measured in mm with a rule. The maximum length will be reported on the CRF and given a code as shown below.

Code	Size
1	< 25 mm
2	25≤ and <50 mm
3	≥ 50 mm

Solicited local reactions will be recorded at visits V2 to V17. The parents/guardians of the volunteers will report on scheduled visits the reactions they may experience, and the field workers will collect information and report on the field worker visit card as scheduled (See flow chart on page 9) after the vaccination.

B. Systemic solicited reactions

The duration in hours or in days of all solicited systemic reaction will be monitored by the investigator, as well as the time interval of occurrence after the last vaccination.

Tympanic temperature will be measured with a digital thermometer and the intensity of fever will be defined as:

Grade	Tympanic temperature
1	38≤ and <38.5 °C
2	38.5≤ and <39 °C
3	≥ 39 °C

The solicited systemic reactions will be monitored at visits V2 to V17. The participants other daily the reactions will be detected on the field worker diary from during field worker visit days.

C. Unsolicited adverse events

All unsolicited adverse events occurring between visits 2-17 shall be documented. If any of them fulfil the criteria of SAE described above in section 6.1.4, they will accordingly be documented and reported.

7.2 `Secondary Evaluation Criteria: Immunogenicity

7.2.1 Humoral Immune response

Definition

The humoral response to the vaccine antigens will be assessed by measuring the level of vaccine-specific IgG.

7.2.1.1 Parameters to be measured

The level of GMZ2-specific IgG will be expressed as amount of IgG bound to the coated antigen. The comparator is a serial dilution of human IgG. A four parameter logistic-regression is fit to the IgG values and the OD-values expressed as amount of IgG bound. A pool of positive and negative control samples is always included in the measurements. Mean ratios with confidence intervals will be given. Four serial dilutions of sera will be done to enhance accuracy of the measurements.

7.2.1.2 Method and Timing of Measurement

The vaccine-specific IgG will be assessed by ELISA for the recombinant proteins GMZ2 expressed in *L. lactis*; rGLURP and rMSP3 expressed in *E. coli*.
The measurements of vaccine-specific IgG resposnses will be performed at baseline, four weeks after each vaccination and one year after the first vaccination (at Day 0, 28, 56, 84 and 365). The final dataset will be generated after the last visit. Interim analyses will be performed if required and used for internal quality control of the final dataset.

7.2.2 Quality of the humoral Immune response:

Definition

The quality of the humoral immune response will be assessed by measuring, IgG1, IgG2, IgG3, IgG4 isotype responses to GMZ2 by ELISA (at Day 0 and 84).

7.2.2.1 Parameters to be measured

The level of vaccine-specific IgG1, IgG2, IgG3 and IgG4 will be expressed as amount of bound Ig.

7.2.2.2 Method and timing of measurement

The vaccine-specific IgG1, IgG2, IgG3 and IgG4 response will be assessed by ELISA for the recombinant proteins rGMZ2 expressed in *L. lactis.*
The measurements will be performed at baseline, and 4 weeks after the third vaccination (Day -14 and 84). Experiments will be performed after Day 84.

7.2.3 Recognition of native antigens of *P. falciparum*

Definition

The quality of the antibodies elicited by the vaccine will be assessed by their ability to recognize the native antigen of air-dried *P. falciparum* mature schizonts.

7.2.3.1 Parameters to be measured

The titre of the antibodies will be expressed as the last serum dilution causing positive fluorescence as compared with non-specific background staining.

7.2.3.2 Method and timing of measurement

The plasma from subjects will be titrated by IFA (Immuno Flourescence Assay) on slides containing *P. falciparum* mature schizonts using two parasite strains.
These measurements will be performed at baseline and four weeks after the second and the third vaccinations (days 0, 56, 84 and 365). All experiments will be performed after blinding in a different laboratory.

7.2.4 Antigen-specific memory B-cells

Definition
Memory B-cell reactivity against GMZ2 will be measured by ELISPOT after stimulation of PBMC with IL-15 and CpG DNA for six days.

7.2.4.1 Parameters to be measured

Spots will be photographed and counted by microscopy by one investigator. Data will be presented as the frequency of antigen-specific B-cells (as percentage of the total IgG-positive memory B-cells per million PBMC).

7.2.4.2 Method and timing of measurement

B-cell ELISPOTs will be performed at Days 0, 84 and 365 on freshly isolated cells.

7.3 Exploratory Evaluation Criteria: Immunogenicity

7.3.1 Vaccine-specific Cellular Immune Response

7.3.1.1 Functionality of the Immune response:

We propose to wait until the Growth Inhibition Assay in the presence of Monocytes (GIM) is validated. Meanwhile, a surrogate marker might be Western blotting. To do this, Vaccine antigens will be resolved using in SDS-PAGE and probed with 1:1000 dilutions of the sera. Bound antibodies are detected by HRP-labelled secondary antibodies and subsequent detection by chemiluminescence.

Definition
The functionality of the antibodies will be assessed by measuring the ability of the antibodies to inhibit the growth *P. falciparum* parasites in the presence and absence of monocytes.

7.3.1.2 Parameters to be measured

The percentage growth inhibition will be determined as follows:
[(% parasitaemia in control wells - % parasitaemia in test wells) x 100]/% parasitaemia in control wells.
The measurement will be done at baseline, after the second and the third dose of vaccination and one year after the first vaccination (at Days -14, 56, 84 and 365).

7.3.1.3 Method and Timing of measurement

An antibody and monocyte dependent growth inhibition assay will be performed as follows: parasites (F32) will be synchronized by sorbitol treatment and allowed to mature to trophozoite/schizont stages (PRBC). PRBC's will be adjusted to 4% haematocrit with culture medium (CM) and to 1% parasitaemia with O+ uninfected erythrocytes. Plasma samples from volunteers will be serially diluted in CM without Albumax II; 100 µl diluted plasma was added to the PRBC suspension in duplicate and incubated at 37°C for 42 hours in the presence or absence of monocytes.

Blood monocytes from healthy non-malaria exposed Swedish volunteers will be separated on Ficoll-Paque and re-suspended in 25% autologous serum. Adherent cells (97% viability) will be removed from a Petri dish after one hour incubation in autologous serum at 37°C in 5% CO2. Monocytes, 1.5x105 to 2x105/well, will be added to the PRBC/plasma suspension and incubated 42 hours at 37°C in 5% CO2. After incubation, cells will be harvested, washed and monolayers (30µl/well) will be prepared and fixed. Parasites will be stained with acridine orange (Fluka Chemie AG, Buchs, and Switzerland) and percentage of only newly infected erythrocytes (ring forms) will be determined per 40.000 erythrocytes in a fluorescence microscope. A serum pool from malaria immune Africans will be used as a positive control and a plasma pool from Swedish blood bank donors as negative control.

7.3.2 Evolution of Vaccine-specific Humoral Immune Response

7.3.2.1 Other antibody classes and modifications:

In addition to established IgG and IgG-subclass-analyses antibody class switching and age-related post-translational modifications are analysed.

Definition

The evolution of antibody classes and post-translational modifications will be assessed by measuring anti-GMZ2 IgM and modified anti-GMZ2 IgG on days -14, 84, and 365.

7.3.2.2 Parameters to be measured

The level of GMZ2-specific Ig classes and modifications will be expressed as amount of Ig bound to the coated antigen. The comparator is a serial dilution of human Ig. A four parameter logistic-regression is fit to the Ig values and the OD-values expressed as amount of Ig bound. A pool of positive and negative control samples is always included in the measurements. Post-translational modifications will be detected by specific antibodies, binding-substrates, and mass-spectrometry.

7.3.2.3 Method and Timing of Measurement

The measurements of vaccine-specific Ig responses will be performed at baseline, four weeks and one year after the first immunisation (at Day -14, 84 and 365). The final dataset will be generated after the last visit.

8 STATISTICAL METHODS AND DATA ANALYSIS

8.1 Principal Objective

To evaluate the safety of three doses of 30 µg and 100 µg GMZ2 adjuvanted with alum hydroxide and given at D0, D28 and D56, in healthy children aged 1-5 years old in Gabon.

8.2 Determination of the Sample Size

The total number of participants will be 30 divided into three groups of 10 each; one receiving 30 µg of GMZ 2, another 100 µg of GMZ2 and the other receiving rabies vaccine (control vaccine). The sample size chosen for this study are in line with similar safety and immunogenicity studies of similar candidate malaria vaccines published in the literature. As a phase I protocol, this study of 30 volunteers is not designed to detect adverse reactions that occur infrequently. Sample size was determined by the requirement to make a preliminary evaluation of inter-group and inter-individual variability, to avoid excessive risk and to allow realistic workload. Statistical significance may not be reached in such a study with low power; however, a non-significant finding would provide justification of the need for a study with greater power.

8.3 Data Set to be analysed

8.3.1 Definition of Population

Given the above mentioned considerations it is planned to enrol 30 participants in the trial, to be randomised into the three treatment arms.
The statistical analysis shall be performed on two types of population depending on the participant's compliance to the protocol:

i). Per Protocol: the vaccination, blood sampling and visits number as well as the time-interval between vaccination and follow-up visits are respected, as they are defined in the protocol and in the flow chart. All participants who have not been compliant with the protocol will be excluded from this analysis but will be described in the final report. The protocol compliance criteria are the following:

- Participants fulfilling the inclusion and exclusion criteria
- Participants who have respected the number of vaccination and the time-intervals between the vaccination:
  - V2: 1st vaccination: D0, interval V1+2 weeks (± 3days)
  - V7: 2nd vaccination D28 , interval V2+4 weeks (± 3 days)
  - V12: 3rd vaccination D56 , interval V5+4 weeks (± 3days)
  - V17: Post vaccination D84 , interval V12+4 weeks (± 7days)
  - V18: Last study visit D365 , interval V17+ 9months (± 14days)
  - Subjects having respected the number of blood samples for biological safety evaluation, as well as the intervals between blood samples on Days -14, 28, 56, 84 and 365 within their applicable intervals

ii). Intention to treat: All participants who have received at least one vaccine dose will be included in the analysis. However, if a participant is not vaccinated for reasons unrelated to the primary outcome measure and the participants' code was broken during the period of the study that participant will be excluded from further analyses. In the event a participant withdraws from the study due to side effects, the last observation will be carried forward in all subsequent analyses (Last Observation Carried Forward LOCF) of the primary outcome measure.

8.4 Population used in analyses

The primary analysis will be conducted on all participants who have received at least one vaccine dose. Participants withdrawing for reasons related to the primary outcome measure, their last observation will be used in all subsequent analyses (LOCF).
Per protocol analysis of the secondary and exploratory outcome measures will be performed on all vaccinated subjects for whom serum samples are available.

8.5 Specific analyses

As mentioned above, the last observation of participants withdrawing for reasons related to the primary outcome measure will be carried forward. The proportion of participants that received three vaccine doses without experiencing side effects will be estimated by the Kaplan-Meier product-limit method.

8.6 Statistical Methods

The analysis shall be descriptive as the sample size does not allow any comparison between groups. For categorical variables, frequency distributions, by vaccination group, will be presented. For continuous variables, box-whisker plots, medians, inter-quartile ranges and ranges will be presented by vaccine group.
The proportion of participants that received three vaccine doses without experiencing (serious) side effects will be estimated by the Kaplan-Meier product-limit method, with time as a discrete variable.

8.6.1 Primary Criterion

The null hypothesis for the primary outcome measure will be evaluated using a one-sample one-sided test for proportions. The null hypothesis states that the frequency of serious adverse reactions is equal among each of the three groups (30 µg GMZ2, 100 µg GMZ2 and rabies vaccinated group). The alternative hypothesis states that this frequency is greater than 25% in the GMZ2 groups.

8.6.2 Secondary Criteria

The secondary and exploratory outcome measures will not be formally analysed with hypothesis testing. The data from the secondary and exploratory outcome measures will be presented as point estimates with 95% confidence intervals. This will enable comparison of the immunogenicity of the three groups at baseline and each of the three vaccinations. As antibody titres usually display skewed distributions, geometric means (with 95% CI) will be calculated instead of arithmetic means.

8.7 Data Management

Throughout regular data collection and monitoring, clinical data reported on CRFs and/or relevant serological/biological sample analysis results scheduled in the protocol will be integrated into the internal clinical data management system.

For each batch of data, double entry, quality control and triggers to computerised logic and/or consistency checks will be systematically applied in order to detect errors or omissions. Moreover, the clinical trial monitor will check through and verify 100% data on CRF with source documents, and all identified errors will be corrected. After integration of all corrections in the complete set of data, the database will be locked and saved before being released for statistical analysis. Each step of this process will be monitored through the implementation of individual passwords and/or regular backups in order to maintain appropriate database access and to guarantee database integrity.

8.7.1 Plans for analysis

A final statistical analysis plan will be agreed upon by the investigators, AMANET and EMVI prior to locking of the database for the final analysis. However, an interim analysis is planned at 28 days post dose 3 (after day 84). This analysis will allow AMANET and EMVI make decisions for the next trial based on the immediate safety profile. This analysis will include information on adverse events by dose and number of vaccinations. Thereafter, the study will continue in a single blind manner for additional safety surveillance and immunogenicity assessment. This additional information will be appended to the study report.

It is anticipated that the results of this study will be presented to the scientific community via oral presentations at meetings and written publications in scientific journals. The data to be presented and the authorship will be discussed between investigators and the sponsor. It is AMANET policy to have the Principal Investigator lead the authorship for work arising from clinical trials, however, the draft manuscript has to be, approved by the sponsor and EMVI prior to any official communication.

The official report of the primary analysis will be submitted through appropriate channels and upon approval by AMANET. This report will contain detailed information about the participants, their tolerance of the vaccines, their side effects and laboratory abnormalities, as well as their overall immune responses to vaccination based on the ICH E-3 and E-9 format.

8.7.2 Source documents and access

Information that cannot be collected initially onto CRFs, namely, clinical laboratory test results and adverse event medical records; screening sheets will first be collected onto separate source documents prior to transcription into CRFs. Only authorized study personnel and study the monitors will have access to CRFs and other source documents, and access to these documents will be controlled by the Clinical Coordinator. The official AMANET source document agreement form which lists the classes of documents will be signed and maintained on the investigator file, including the home visit follow-up diary cards.

8.7.3 Data ownership

The results emanating from this study primarily belongs to the AMANET/EMVI. However, a consortium comprising the investigators, AMANET (study sponsor), and EMVI (product owner) shall corporately work towards development and licensure of GMZ2.

9 SITE MONITORING PLAN

Site monitoring will be conducted by a qualified and experienced clinical monitor appointed by AMANET to ensure that ICH-GCP standards and regulatory guidelines are being followed. Pre-trial monitoring visits will be made to the site, including the clinical laboratory. All records will be made available to monitors, including regulatory files, CRFs and other source documents, QA/QC documentation, SOPs, etc. An agreed site monitoring schedule will be reached before study start, but at the discretion of the monitor, additional site visits may be made during the course of the trial and at the end of the surveillance period.

The monitoring plan will include the number of participant charts to be reviewed, which/what proportion of data fields and what will be monitored, and who will be responsible for conducting the monitoring visits, and who will be responsible for ensuring that monitoring findings are addressed.

Before each monitoring visit, the monitor will contact the principal investigator and send a proposed visit agenda for discussion. During the visit, the monitor will undertake 100% source document verification, check all informed consent documents, and inspect site facilities, state of equipment maintenance, vaccine accountability and specimen storage documents, and general compliance of investigators to ICH-GCP.

For each visit, a detailed monitoring report on the standard forms shall be provided within two weeks, together with a specific action items summary. This report will be reviewed by two representatives from GMZ2 Clinical Development Team and any issues arising will accordingly be followed up.

10 QUALITY CONTROL AND QUALITY ASSURANCE

10.1 QA/QC Policy

SOPs for quality management which have been inexistence and use will be reviewed and updated and used to train appropriate personnel, and kept on file with documentation of the training. Data will be evaluated for compliance with protocol and accuracy in relation to source documents. The study will be conducted in accordance with procedures identified in the protocol. The types of materials to be reviewed, who are responsible, and the schedule for reviews will be referenced in the SOPs. Study-specific training will be provided for all staff prior to the commencement of the trial.
Regular monitoring will be performed according to ICH-GCP. Following written SOPs, the monitors will verify that the clinical trial is conducted and data are generated, documented (recorded), and reported in compliance with the protocol, ICH-GCP, and the applicable regulatory requirements. Reports will be submitted to AMANET on monitoring activities and specific recommendations will be implemented to improve the quality of the study. The MRU at Lambaréné will implement quality control procedures beginning with form design, training, tracking system, data entry system and generate data quality control checks that will be run on the database. Any missing data or data anomalies will be communicated to the field site for clarification/resolution.

The investigational site will provide direct access to all trial related source data/documents, and reports for the purpose of monitoring and auditing by the sponsor, and inspection by local and regulatory authorities as the case may be.

10.2 Modification/Amendment of the Protocol

No amendments to this protocol will be made without consultation with, and agreement of, the sponsor. Any changes that appear necessary during the course of the trial must be discussed by the investigator and sponsor concurrently. If agreement is reached concerning the need for an amendment, it will be produced in writing by the sponsor and/or the investigator and will be made a formal part of the protocol. An amendment may require IEC/IRB approval. All amendments must also be transmitted to Regulatory Authorities, if applicable.
An administrative change to the protocol is one that modifies administrative and logistical aspects of a protocol but does not affect the subjects' safety, the objectives of the trial and its progress. An administrative change does not require IEC/IRB approval. However, the IEC/IRB must be notified whenever an administrative change is made. All amendments involving a change in the study procedure must seek for approval. All amendments involving a change in the study procedure must seek for approval.
The investigator is responsible for ensuring that changes to an approved trial, during the period for which IEC/IRB approval has already been given, is not initiated without IEC/IRBs review and written approval except to eliminate apparent immediate hazards to the subject

10.3 Archiving

The investigator must keep all trial documents provided by AMANET for at least 15 years after the completion or discontinuation. The investigator will inform AMANET should there be need for any changes. In the event of lack of storage capacity, the investigator shall report to the

sponsor to make specific archival arrangement according to sponsor SOPs and applicable regulatory requirements.

11 ETHICS/PROTECTION OF HUMAN PARTICIPANTS

11.1 Ethical Standard

The Independent Regional Ethics Committee of Lambaréné (CERIL) is a legally mandated entity by the Gabon government to review and approve research involving human subjects. This protocol will be approved by the Scientific Committee of AMANET before submission to the local authorities.

Documentation of the approval by the ethical and scientific review boards will be kept in both the investigator and sponsor's study files. Moreover, the study vaccines will only be shipped to the site after AMANET acknowledges receipt of the local ethical approval. This trial will uphold the standards as articulated in the major ICH-GCP and ethical guidelines. To ensure ethical conduct of the trial, all the clinical investigators will be required to have a certificate of research ethics training at a minimum.

The investigators will inform all the IEC/IRBs and AMANET of the following:

- All subsequent protocol amendments, informed consent changes or revisions of other documents originally submitted for review
- Serious and/or unexpected adverse events occurring during the study, where required
- New information, that may affect adversely the safety of the participants or the conduct of the study
- An annual update and/or request for re-approval, where required
- When the study has been completed.

11.2 Informed Consent Process

The principles of informed consent in the current edition of the Declaration of Helsinki will be implemented before any protocol-specified procedures or interventions are carried out.
Information will be given in both oral and written form whenever possible. Independent witnesses will be required to attest that illiterate guardians of potential participants have understood the contents of the informed consent.

11.3 Screening and study informed consent

Informed consent is considered to be a dynamic, ongoing process, with continuous availability of investigators to answer any questions that arise in the course of the trial and to ensure that parents/guardians of the participants understand trial procedures. Should new data become available that could affect participant safety and/or willingness to continue in the study, informed consent would be obtained and documented again.
The extensive contact between the team of investigators and the population of Lambaréné has led to the development of mutual trust and the establishment of an ongoing informed consent process attempting to address issues related to interventional studies in resource-limited settings. Many discussions with local community leaders, heads of families and citizens through group meetings, and more limited group interviews will be undertaken prior to study start.
Once the group of community leaders has expressed their approval of the planned study, information will be disseminated to various constituencies, so that when potential recruits are approached by study staff they are already generally aware of the nature of the impending

study. We do not consider this process to constitute “community consent” in addition to or in lieu of individual informed consent, but rather a community “permission to enter” that is a necessary prerequisite to conducting any study in a tight-knit and highly organized traditional rural community.
A period of approximately four weeks is allotted for screening and recruitment to allow enough time for parents/guardians of potential participants’ to consider their decision about participating and to discuss their participation with family members and others in the community. At the times of screening and recruitment, the form will be given to parents/guardians of the participants who speak French, and translated orally into the local dialect of each participant who is illiterate. All relevant information on the participant responsibilities including the possible risks such as discomfort and pain at injections, the clinic visits they need to honour etc, will be explained. In all cases, the investigator will give the participants ample opportunity to inquire about the details of the study and to ask any questions before dating and signing the consent forms, including the opportunity to take a copy of the consent form home to review with family members or others before returning on a later day with their decision. All illiterate parents/guardians will have the study and consent forms explained to them point by point by the interviewer in the presence of a witness who will sign the consent form. The witnesses will have no association with the conduct of the study and will not be related to the study participant; efforts will be made to secure confidentiality for participants.
Informed consent will be documented by the use of a written form approved by the IEC/IRB, signed or thumb-printed and dated by the participant’s legal guardian and by the person who conducted the informed consent discussion. Thumb printing will be used for illiterate persons only, who are expected to constitute the majority of participants. The signature/thumbprint confirms that the consent is based on information that has been understood. Each parents/guardians of participant’s signed informed consent form will be kept on file by the investigator for checking by the clinical monitor and possible inspection by regulatory authorities. The participant will receive a copy of the signed and dated written informed consent forms and any other written information provided by the investigator, and will receive copies of any signed and dated consent form updates and any amendments to the written information.
In case of study participants without access telephones, fax or mail, contact information will be provided for physicians on the investigator team who can be visited directly.

11.4 Participant Confidentiality

Participant confidentiality will strictly be up held in trust by the participating investigators, their staff, and the sponsors and their agents. This confidentiality is extended to cover testing of biological samples and genetic tests in addition to the clinical information relating to participating participants. Before study start, all members of staff on the investigator team will sign an agreement to this effect.
The study protocol, documentation, data, and all other information generated will be held in strict confidence. No information concerning the study or the data will be released to any unauthorised third party without prior written approval of the sponsors.
The study monitor or other authorised representatives of the sponsors may inspect all documents and records required to be maintained by the investigator, including but not limited to, medical records (office, clinic, or hospital) and pharmacy records for the participants in this study. The clinical study site will permit access to such records.

11.5 Future Use of Stored Specimens

The residual sera and cells remaining after the serological and CMI assays described in this protocol, may be used for additional immunological and in vitro studies related to malaria. Permission will be expressly granted for preserving samples for future studies at the time of informed consent at study enrolment. To ensure appropriate storage, the samples will be shipped to Denmark in custody of the EMVI. A material transfer agreement proposed by the MRU will be reached among the consortium members, which may be extended to efforts of the European Malaria Vaccine Development work.

11.6 Publication Policy

According to AMANET policy, all clinical trials must be registered with an appropriate authority before commencement of the study. This protocol will be registered with the US ClinicalTrials.gov registry and the relevant registration number NCT updated once obtained.
The Principal Investigator, in collaboration with the Clinical Trials Coordinator at AMANET, and the Clinical Operations Manager at EMVI will prepare the final report. It will be signed by the Principal Investigator. The protocol and data derived from the trial are the exclusive property of AMANET and EMVI. Any publication or presentation related to the trial must be approved by AMANET/EMVI before submission of the manuscript. The Principal Investigator is expected to lead the process of peer reviewed journal publication, and this must be done within six months of completing the clinical study report.
AMANET/EMVI must have the opportunity to review all proposed abstracts, manuscripts or presentations regarding this trial at least 30 days prior to submission for publication/ presentation. AMANET has to be acknowledged in any publication arising from this protocol.

On Authorship, AMANET requires that all those listed meet the criteria for authorship as outlined by the International Committee of Medical Journal Editors (ICMJE). Every individual listed as author of the publication(s) must have contributed to the protocol and/or to the analysis of the data, and should be able to defend the manuscript.

12. Literature cited

(1) Snow RW, Craig M, Deichmann U, Marsh K. Estimating mortality, morbidity and disability due to malaria among Africa's non-pregnant population. Bull World Health Organ **1999**;77(8):624-40.

(2) Alonso PL, Armstrong JR, Lindsay SW. Malaria, bednets, and mortality. Lancet **1991**;338(8771):897.

(3) Nevill CG, Some ES, Mung'ala VO, et al. Insecticide-treated bednets reduce mortality and severe morbidity from malaria among children on the Kenyan coast. Trop Med Int Health **1996**;1(2):139-46.

(4) D'Alessandro U, Olaleye BO, McGuire W, et al. Mortality and morbidity from malaria in Gambian children after introduction of an impregnated bednet programme. Lancet **1995**;345(8948):479-83.

(5). Sylla EH, Kun JF, Kremsner PG. Mosquito distribution and entomological inoculation rates in three malaria-endemic areas in Gabon. Trans R Soc Trop Med Hyg. 2000; 94:652-6.

(6). Wildling E, Winkler S, Kremsner PG, Brandts C, Jenne L, Wernsdorfer WH. Malaria epidemiology in the province of Moyen Ogooue, Gabon. Trop Med Parasitol. 1995; 46:77-82.

(7). Sylla EH, Lell B, Kun JF, Kremsner PG. Plasmodium falciparum transmission intensity and infection rates in children in Gabon. Parasitol Res. 2001; 87:530-3.

(8). Borrmann S, Binder RK, Adegnika AA, Missinou MA, Issifou S, Ramharter M, Wernsdorfer WH, Kremsner PG. Reassessment of the resistance of Plasmodium falciparum to chloroquine in Gabon: implications for the validity of tests in vitro vs. in vivo. Trans R Soc Trop Med Hyg. 2002; 96:660-3.

(9.) Taylor T, Olola C, Valim C, Agbenyega T, Kremsner P, Krishna S, Kwiatkowski D, Newton C, Missinou M, Pinder M, Wypij D. Standardized data collection for multi-center clinical studies of severe malaria in African children: establishing the SMAC network. Trans R Soc Trop Med Hyg. 2006; 100:615-22.

(10.) Lell B, May J, Schmidt-Ott RJ, Lehman LG, Luckner D, Greve B, Matousek P, Schmid D, Herbich K, Mockenhaupt FP, Meyer CG, Bienzle U, Kremsner PG. The role of red blood cell polymorphisms in resistance and susceptibility to malaria. Clin Infect Dis. 1999; 28:794-9.

(11) Cerami C, Frevert U, Sinnis P, et al. The basolateral domain of the hepatocyte plasma membrane bears receptors for the circumsporozoite protein of Plasmodium falciparum sporozoites. Cell **1992**;70(6):1021-33.

(14) Vreden SG, Verhave JP, Oettinger T, Sauerwein RW, Meuwissen JH. Phase I clinical trial of a recombinant malaria vaccine consisting of the circumsporozoite repeat region of Plasmodium falciparum coupled to hepatitis B surface antigen. Am J Trop Med Hyg **1991**;45(5):533-8.

(15) Alonso PL, Sacarlal J, Aponte JJ, et al. Efficacy of the RTS,S/AS02A vaccine against Plasmodium falciparum infection and disease in young African children: randomised controlled trial. Lancet **2004**;364(9443):1411-20.

(16) Alonso PL, Sacarlal J, Aponte JJ, et al. Duration of protection with RTS,S/AS02A malaria vaccine in prevention of Plasmodium falciparum disease in Mozambican children: single-blind extended follow-up of a randomised controlled trial. Lancet **2005**;366(9502):2012-8.

(17) Good MF, Kaslow DC, Miller LH. Pathways and strategies for developing a malaria blood-stage vaccine. [Review] [94 refs]. Annual Review of Immunology **1998**;16:57-87.

(18) Narum DL, Thomas AW. Differential localization of full-length and processed forms of PF83/AMA-1 an apical membrane antigen of Plasmodium falciparum merozoites. Mol Biochem Parasitol **1994**;67(1):59-68.

(19) Crewther PE, Culvenor JG, Silva A, Cooper JA, Anders RF. Plasmodium falciparum: two antigens of similar size are located in different compartments of the rhoptry. Exp Parasitol **1990**;70(2):193-206.

(20) Waters AP, Thomas AW, Deans JA, et al. A merozoite receptor protein from Plasmodium knowlesi is highly conserved and distributed throughout Plasmodium. J Biol Chem **1990**;265:17974-9.

(21) Kocken CH, van der Wel AM, Dubbeld MA, et al. Precise timing of expression of a Plasmodium falciparum-derived transgene in Plasmodium berghei is a critical determinant of subsequent subcellular localization. J Biol Chem **1998**;273(24):15119-24.

(22) Deans JA, Alderson T, Thomas AW, Mitchell GH, Lennox ES, Cohen S. Rat monoclonal antibodies which inhibit the in vitro multiplication of Plasmodium knowlesi. Clin Exp Immunol **1982**;49(2):297-309.

(23) Thomas AW, Deans JA, Mitchell GH, Alderson T, Cohen S. The Fab fragments of monoclonal IgG to a merozoite surface antigen inhibit Plasmodium knowlesi invasion of erythrocytes. Mol Biochem Parasitol **1984**;13(2):187-99.

(24) Peterson MG, Marshall VM, Smythe JA, et al. Integral membrane protein located in the apical complex of Plasmodium falciparum. Mol Cell Biol **1989**;9(7):3151-4.

(25) Waters AP, Thomas AW, Deans JA, et al. A merozoite receptor protein from Plasmodium knowlesi is highly conserved and distributed throughout Plasmodium. J Biol Chem **1990**;265(29):17974-9.

(26) Marshall VM, Zhang L, Anders RF, Coppel RL. Diversity of the vaccine candidate AMA-1 of Plasmodium falciparum. Mol Biochem Parasitol **1996**;77(1):109-13.

(27) Oliveira DA, Udhayakumar V, Bloland P, et al. Genetic conservation of the Plasmodium falciparum apical membrane antigen-1 (AMA-1). Mol Biochem Parasitol **1996**;76(1-2):333-6.

(22) Hodder AN, Crewther PE, Matthew ML, et al. The disulfide bond structure of Plasmodium apical membrane antigen-1. J Biol Chem **1996**;271(46):29446-52.

(23) Thomas AW, Trape JF, Rogier C, Goncalves A, Rosario VE, Narum DL. High prevalence of natural antibodies against Plasmodium falciparum 83-kilodalton apical membrane antigen (PF83/AMA-1) as detected by capture-enzyme-linked immunosorbent assay using full-length baculovirus recombinant PF83/AMA-1. Am J Trop Med Hyg **1994**;51(6):730-40.

(24) Udhayakumar V, Kariuki S, Kolczack M, et al. Longitudinal study of natural immune responses to the Plasmodium falciparum apical membrane antigen (AMA-1) in a holoendemic region of malaria in western Kenya: Asembo Bay Cohort Project VIII. Am J Trop Med Hyg **2001**;65(2):100-7.

(26) Hodder AN, Crewther PE, Anders RF. Specificity of the protective antibody response to apical membrane antigen 1. Infect Immun **2001**;69(5):3286-94.

(27) Cortes A, Mellombo M, Mueller I, Benet A, Reeder JC, Anders RF. Geographical structure of diversity and differences between symptomatic and asymptomatic infections for Plasmodium falciparum vaccine candidate AMA1. Infect Immun **2003**;71(3):1416-26.

(28) Kennedy MC, Wang J, Zhang Y, et al. In vitro studies with recombinant Plasmodium falciparum apical membrane antigen 1 (AMA1): production and activity of an AMA1 vaccine and generation of a multiallelic response. Infect Immun **2002**;70(12):6948-60.

(29) Kocken CH, Withers-Martinez C, Dubbeld MA, et al. High-level expression of the malaria blood-stage vaccine candidate Plasmodium falciparum apical membrane antigen 1 and induction of antibodies that inhibit erythrocyte invasion. Infect Immun **2002**;70(8):4471-6.

(30) Healer J, Murphy V, Hodder AN, et al. Allelic polymorphisms in apical membrane antigen-1 are responsible for evasion of antibody-mediated inhibition in Plasmodium falciparum. Mol Microbiol **2004**;52(1):159-68.

(31) Plowe CV, Thera MA, Takala SL, et al. Dynamics of *Plasmodium falciparum* apical membrant antigen-1 sequence variation over three years at a malaria vaccine testing site in Bandiagara, Mali. 73 ed. **2005**. p. 277.

(32) Gordon DM, McGovern TW, Krzych U, et al. Safety, immunogenicity, and efficacy of a recombinantly produced Plasmodium falciparum circumsporozoite protein-hepatitis B surface antigen subunit vaccine. J Infect Dis **1995**;171(6):1576-85.

(33) Wu JY, Gardner BH, Murphy CI, et al. Saponin adjuvant enhancement of antigen-specific immune responses to an experimental HIV-1 vaccine. J Immunol **1992**;148(5):1519-25.

(34) Soltysik S, Bedore DA, Kensil CR. Adjuvant activity of QS-21 isomers. Ann N Y Acad Sci **1993**;690:392-5.

(35) Ribi E, Cantrell J, Feldner T. Microbiology. Washington, DC: American Society of Microbiology, **1986**.

(36) Myers KR, Truchot AT, Ward J. Cellular and molecular aspects of endotoxin reactions. In: Nowotny A, ed.Amsterdam: **1990**. p. 145-56.

(37) Loppnow H, Durrbaum I, Brade H, et al. Lipid A, the immunostimulatory principle of lipopolysaccharides? Adv Exp Med Biol **1990**;256:561-6.

(38) Johnson AG, Tomai MA. A study of the cellular and molecular mediators of the adjuvant action of a nontoxic monophosphoryl lipid A. Adv Exp Med Biol **1990**;256:567-79.

(39) Doherty JF, Pinder M, Tornieporth N, et al. A phase I safety and immunogenicity trial with the candidate malaria vaccine RTS,S/SBAS2 in semi-immune adults in The Gambia. Am J Trop Med Hyg **1999**;61(6):865-8.

(40) Bojang KA, Milligan PJ, Pinder M, et al. Efficacy of RTS,S/AS02 malaria vaccine against Plasmodium falciparum infection in semi-immune adult men in The Gambia: a randomised trial. Lancet **2001**;358(9297):1927-34.

(41) Bojang KA, Olodude F, Pinder M, et al. Safety and immunogenicty of RTS,S/AS02A candidate malaria vaccine in Gambian children. Vaccine **2005**;23(32):4148-57.

(42) Stoute JA, Gombe J, Withers MR, et al. Phase 1 randomized double-blind safety and immunogenicity trial of Plasmodium falciparum malaria merozoite surface protein FMP1 vaccine, adjuvanted with AS02A, in adults in western Kenya. Vaccine **2005**.

(43) Dreesen DW, Fishbein DB, Kemp DT, Brown J. Two-year comparative trial on the immunogenicity and adverse effects of purified chick embryo cell rabies vaccine for pre-exposure immunization. Vaccine **1989**;7(5):397-400.

(44) Nicholson KG, Farrow PR, Bijok U, Barth R. Pre-exposure studies with purified chick embryo cell culture rabies vaccine and human diploid cell vaccine: serological and clinical responses in man. Vaccine **1987**;5(3):208-10.

(45) Vodopija I, Sureau P, Lafon M, et al. An evaluation of second generation tissue culture rabies vaccines for use in man: a four-vaccine comparative immunogenicity study using a pre-exposure vaccination schedule and an abbreviated 2-1-1 postexposure schedule. Vaccine **1986**;4(4):245-8.

(46) Wasi C, Chaiprasithikul P, Chavanich L, Puthavathana P, Thongcharoen P, Trishanananda M. Purified chick embryo cell rabies vaccine. Lancet **1986**;1(8471):40.

(47) Cox JH, Schneider LG. Prophylactic immunization of humans against rabies by intradermal inoculation of human diploid cell culture vaccine. J Clin Microbiol **1976**;3(2):96-101.

(48) Kuwert EK, Marcus I, Werner J, Iwand A, Thraenhart O. Some experiences with human diploid cell strain-(HDCS) rabies vaccine in pre- and post-exposure vaccinated humans. Dev Biol Stand **1978**;40:79-88.

(49) Ajjan N, Soulebot JP, Stellmann C, et al. [Results of preventive rabies vaccination with a concentrated vaccine of the PM/WI38-1503-3M rabies strain cultured on human diploid

cells. Preparation of mixed antirabies-antitetanus hyperimmune immunoglobulin by plasmapheresis of blood taken from vaccinated veterinary students]. Dev Biol Stand **1978**;40:89-100.

(50) Costy-Berger F. [Preventive rabies vaccination using vaccine prepared from human diploid cells]. Dev Biol Stand **1978**;40:101-4.

(51) Dreesen DW. A comparative study of cell culture rabies vaccine: Immunogenicity and safety. Protocol 8USA301RA, 1985 - 1986. **1986**.

(52) Lyke KE, Diallo DA, Dicko A, et al. Association of intraleukocytic Plasmodium falciparum malaria pigment with disease severity, clinical manifestations, and prognosis in severe malaria. Am J Trop Med Hyg **2003**;69(3):253-9.

(53) Lyke KE, Burges R, Cissoko Y, et al. Serum Levels of the Proinflammatory Cytokines Interleukin-1 Beta (IL-1{beta}), IL-6, IL-8, IL-10, Tumor Necrosis Factor Alpha, and IL-12(p70) in Malian Children with Severe Plasmodium falciparum Malaria and Matched Uncomplicated Malaria or Healthy Controls. Infect Immun **2004**;72(10):5630-7.

(54) Djimde A, Doumbo OK, Cortese JF, et al. A molecular marker for chloroquine-resistant falciparum malaria. N Engl J Med **2001**;344(4):257-63.

(55) Dvorak,J.A., Miller,L.H., Whitehouse,W.C. & Shiroishi,T. Invasion of erythrocytes by malaria merozoites. *Science* 187, 748-750 **(1975**).

(56) Sodiomon B Sirima, et al. Safety and immunogenicity of the plasmodium falciparum merozoite surface protein-3 long synthetic peptide (MSP 3 LSP) malaria vaccine in healthy, semi immune males in Burkina Faso, West Africa. Vaccine; JVAC6330-10. 2006.
